# Patterns of Diversity in Soft-Bodied Meiofauna: Dispersal Ability and Body Size Matter

**DOI:** 10.1371/journal.pone.0033801

**Published:** 2012-03-23

**Authors:** Marco Curini-Galletti, Tom Artois, Valentina Delogu, Willem H. De Smet, Diego Fontaneto, Ulf Jondelius, Francesca Leasi, Alejandro Martínez, Inga Meyer-Wachsmuth, Karin Sara Nilsson, Paolo Tongiorgi, Katrine Worsaae, M. Antonio Todaro

**Affiliations:** 1 Dipartimento di Zoologia e Genetica Evoluzionistica, Università di Sassari, Sassari, Italy; 2 Centre for Environmental Sciences, Hasselt University, Diepenbeek, Belgium; 3 Department of Biology, University of Antwerp, Wilrijk, Belgium; 4 Department of Invertebrate Zoology, Swedish Museum of Natural History, Stockholm, Sweden; 5 Division of Biology, Imperial College London, Ascot, United Kingdom; 6 Dipartimento di Biologia, Universtità di Modena e Reggio Emilia, Modena, Italy; 7 Marine Biological Section, University of Copenhagen, Helsingør, Denmark; Université du Québec à Rimouski, Canada

## Abstract

**Background:**

Biogeographical and macroecological principles are derived from patterns of distribution in large organisms, whereas microscopic ones have often been considered uninteresting, because of their supposed wide distribution. Here, after reporting the results of an intensive faunistic survey of marine microscopic animals (meiofauna) in Northern Sardinia, we test for the effect of body size, dispersal ability, and habitat features on the patterns of distribution of several groups.

**Methodology/Principal Findings:**

As a dataset we use the results of a workshop held at La Maddalena (Sardinia, Italy) in September 2010, aimed at studying selected taxa of soft-bodied meiofauna (Acoela, Annelida, Gastrotricha, Nemertodermatida, Platyhelminthes and Rotifera), in conjunction with data on the same taxa obtained during a previous workshop hosted at Tjärnö (Western Sweden) in September 2007. Using linear mixed effects models and model averaging while accounting for sampling bias and potential pseudoreplication, we found evidence that: (1) meiofaunal groups with more restricted distribution are the ones with low dispersal potential; (2) meiofaunal groups with higher probability of finding new species for science are the ones with low dispersal potential; (3) the proportion of the global species pool of each meiofaunal group present in each area at the regional scale is negatively related to body size, and positively related to their occurrence in the endobenthic habitat.

**Conclusion/Significance:**

Our macroecological analysis of meiofauna, in the framework of the ubiquity hypothesis for microscopic organisms, indicates that not only body size but mostly dispersal ability and also occurrence in the endobenthic habitat are important correlates of diversity for these understudied animals, with different importance at different spatial scales. Furthermore, since the Western Mediterranean is one of the best-studied areas in the world, the large number of undescribed species (37%) highlights that the census of marine meiofauna is still very far from being complete.

## Introduction

Due to their taxonomic diversity and species richness, microscopic animals (meiofauna) represent an important but often neglected component of marine biodiversity [1]. Moreover, to date large-scale taxonomic surveys of the actual contribution of these organisms to local diversity and analyses of their correlates of diversity have rarely been attempted. This is an unfortunate situation: most of the marine biodiversity may reside in meiofauna, but their actual diversity is unknown and so it is impossible to infer the drivers of diversity in the group. Additionally, most of the animal phyla are represented in the meiofauna [2]; therefore, suites of organisms from completely different evolutionary histories are present in the same habitats, providing an invaluable tool to identify generalities in macroecology and biogeography, regardless of phylogenetic constraints.

The aim of this research is twofold. First, we provide an annotate checklist of soft-bodied meiofauna from a marine protected area of the Western Mediterraneans sea, a region recognized as a marine biodiversity hotspot [3]. Second, we perform the first analysis on the ecological and biological correlates of patterns of diversity in marine meiofauna in a macroecological framework. Such analysis may be able to shed light on the generality of the processes governing biodiversity in our changing world.

The faunistic survey was carried out at La Maddalena Marine National Park (Northern Sardina, Italy) in September 2010 in the course of a 10-day workshop during which the following taxa were studied: Acoela, Annelida, Gastrotricha, Nemertodermatida, Platyhelminthes and Rotifera. It should be highlighted that taxonomical work on these animals relies on observations on live material; consequently, the current knowledge on them is particularly poor.

The macroecological analyses, in addition to the Sardinian data, include data obtained on the same taxonomic groups during a previous workshop held in September 2008 in Western Sweden. The Swedish workshop was hosted at The Sven Lovén Centre for Marine Sciences on the island of Tjärnö and saw the participation of experts of most meiobenthic taxa including most of the authors [4]. In a two-week period in Tjärnö, 430 species of meiofauna were found, with the discovery of 157 species new to Sweden and 27 new to science. By analysing two data sets using identical methods but covering different biogeographical areas (Northern Sardinia+Western Sweden), we are able to search for generalities. If generalities are present in the macroecological processes driving diversity in the different groups of meiofauna, we should observe similar patterns in the two sampling areas, notwithstanding the eco-physiographic differences between them. Alternatively, if correlates of diversity are different between the two areas, we can infer that local forces outcompete global drivers in producing patterns of diversity in meiofauna.

According to the ubiquity theorem, microscopic organisms are more widely distributed than larger ones, and the proportion of local species richness to the global species pool is negatively related to body size [5]; thus, the probability to find new species with restricted distribution should be lower in smaller than in larger organisms. Such a strong relationship between body size and biodiversity patterns may be a misrepresentation of reality, and other features of the organisms themselves or of the environment may play a major role in driving diversification and distribution in space [6]. Thus, we used the two highly detailed faunistic lists of different phyla of marine meiofauna from Northern Sardinia and from Western Sweden to address the issue of the importance of body size and other correlates of diversity, using linear mixed effects models (LMEMs) to account for potential pseudoreplication [7], and model averaging [8] to asses the importance of the potential correlates.

## Materials and Methods

### Study Areas

#### Northern Sardinia

The sampling area is located in the Strait of Bonifacio, between Sardinia and Corsica (Western Mediterranean Sea; see Figure S1). Water circulation in the Strait is strongly controlled by winds: current intensity varies between 0.10 and 0.50 m/s, with higher values in shallower areas during the prevailing N-W Mistral wind [9]. Water temperature varies from 15°C in early spring to 25°C in summer. Salinity in the area is constant during the year, ranging 37.7–38.8‰. Maximum tidal range is about 0.25 m [10].

The strong hydrodynamics and the presence of extensive *Posidonia oceanica* seagrass meadows, down to a depth of ≈40 m, influence the sediments in the area, which show a gravelly-sand composition, with mud content generally <5%, and a high CaCo_3_ content, with maximum values >75% [9]. Locally, less sever hydrodynamic conditions favour the presence of small sandy beaches that in good number characterize the coastline of the islands.

#### Western Sweden

The investigated areas is located in northern Skagerrak on the border between Norway and Sweden. Tidal amplitudes are 0.1–0.4 m. The water circulation is largely determined by winds and large-scale currents. There is a marked seasonality in the water temperature: winter surface temperatures may reach the freezing point and there is frequently ice formation, whereas summer surface temperatures may exceed 20°C. Salinity in the surface layer down to 20 m is affected by currents and precipitation. It varies between 10 and 34‰. The sediments are generally mixed, ranging from fine mud to coarse gravel. There are deepwater *Lophelia* coral reefs in the northern part of the area. Numerous islands and islets provide exposed as well as sheltered conditions; sandy coves and sandbars are present in many places.

### Sampling

#### Northern Sardinia

Samples were collected between September 5^th^ and 15^th^, 2010; most of them were collected from the islands forming the archipelago of La Maddalena; additional samples where collected from stations located along the northwestern coast of Sardinia, i.e. Costa Paradiso and Capo Caccia (Figure S1). The investigated habitats ranged from littoral beaches and rock pools to sublittoral sediments to about −37 m, including marine caves. Samples from this area consisted mostly of clean fine and coarse sand, without mud of silt. Littoral samples were taken by hand or with a plankton net, sublittoral samples were taken by scuba divers. Detailed information on sampling localities is given in Table S1.

#### Western Sweden

Samples were collected between September 2^nd^ and 13^th^, 2007 mostly around the island of Tjärnö (Koster archipelago); the sampled habitats ranged from littoral beaches and rock pools to sublittoral mudflats, mostly at depths between 0 and −38 m. Littoral samples were mostly taken by hand or a plankton-net whereas sublittoral samples were taken by boat using a dredge or Warén sledge. The majority of the sediment samples were rich in silt and mud, and even sandy samples had a strong component of silt, with some noticeable exceptions. Details on sampling techniques and characteristics the sampling sites can be found in Willems et al. [4].

Except for sampling within La Maddalena Marine National Park (Ente Parco-protocollo/permit n. 2768/11), no special permission/permits were needed to collect these animals, because meiofauna are microscopic, non-pathogenic animals, field study did not involve endangered species and sampling was carried out in public beaches. Moreover, no meiofauna species are under special conservation concerns.

### Sample and organism processing

During both workshops, samples were taken to the laboratory soon after collection and processed within few days. Specimens were extracted daily using two different methods: both by MgCl_2_-decantation and by siphoning off the water just above the sediment [2], [4]. Algae samples were rinsed with MgCl_2_. Live material was studied using dissecting and light microscopes. Additional material for identification and/or descriptive purposes was preserved using methods appropriate for the respective taxon [11]–[16].

A detailed description of the faunistic results from the Sardinian workshop is provided in the first part of the results section; a summary list of the soft-bodied meiofaunal taxa found during the Swedish workshop is provided in Table S2 while exhaustive information can be found in Willems et al [4].

### Statistical analyses

Taking advantage of the robust and comparable datasets offered by the two workshops, we aimed at identifying the relevant correlates of the diversity patterns in meiofaunal organisms. We used linear mixed effects models [17] and model averaging [8] to investigate the effect and the importance of a set of biological and environmental variables as predictors of different response variables describing different facets of biological diversity. We implemented four separate statistical analyses, each one using a different response variable, accounting for geographic range size, number of new species unknown to science, and ratio of regional to global and of local to regional species richness, explained in details below from (i) to (iv). As explanatory variables, we used the ones that could be ecologically relevant and we obtained estimates for six variables (explained in details below, from 1 to 6), from measurements taken from the organisms we collected, and/or from the literature. Both the explanatory and the response variables were measured separately for the two surveys in Northern Sardinia and Western Sweden.

#### Explanatory variables

They accounted for biological (body size, dispersal potential, reproductive mode) and environmental variables (habitat, substrate and depth). An estimate of (1) body size (median body length) for each species was obtained from the adult individuals collected in the field and/or from literature data (Table S2). To estimate the (2) potential for dispersal, we collected information on presence/absence of resting or dispersing stages (Table S2); for the (3) reproductive mode, we categorised organisms as exclusively parthenogenetic or not (Table S2). To estimate environmental variables, we used three different metrics: (4) habitat specificity, (5) kind of substrate and (6) depth. For habitat specificity, species were grouped as exclusively endobenthic (living only in the sediments, either as interstitial or borrower) or not (Table S2); for kind of substrate, we identified 18 categories depending on the type of sample (e.g. sediments with different granulometry such as pebble, coarse sand, medium sand, fine sand, mud, silt, or other habitats such as periphyton, epibiont, etc.); as for depth, the measured depth of the sample was used (Table S1) and [4]. Then, we obtained summary statistics for these six variables for six taxonomic groups (Acoela, Annelida, Gastrotricha, Proseriata, Rhabdocoela and Rotifera), whereas Nemertodermatida were not included, due to the taxonomic uncertainties in the group and the paucity of information in the literature. For each variable we calculated the following summary statistics, separately for Western Sweden and Northern Sardinia: for body size, the median value of all species for each group (no measure of variability was included, because the coefficient of variation, standard deviation/mean, was well below 1 in all cases, except for annelids in Sardinia and rotifers in Sweden); for dispersal potential, the proportion of species with resting or dispersing stages; for reproductive mode, the proportion of species with parthenogenetic reproduction; for habitat specificity, the proportion of exclusively endobenthic species; for kind of habitat, the proportion of types of habitat where each group was found, in comparison to the total number of types; for depth, the depth range for each group, in comparison to the overall depth range.

#### Response variables

Different aspects of biodiversity for each of the six taxonomic groups could be influenced by the explanatory variables that we assessed; we included four different response variables for four different theoretical rationales in our models, listed below from (i) to (iv).

Geographic distribution of animals is a function of ecological and historical variables; thus, we tested whether the (i) geographic range size of the different taxonomic groups was influenced by the six ecological variables we measured. To do so, we grouped the species we found according to whether they have wide or limited biogeographical range, limited to the Mediterranean (for the Sardinian dataset) or to the North Sea – Baltic area (for the Swedish dataset); we then used the proportion of species with limited range as a response variable. The expectation is that smaller organisms with high dispersal potential are more widely distributed than larger ones without dispersing stages.

We acknowledge that this analysis could be biased by the large amount of unknown diversity in meiofauna; thus, we applied also an alternative rationale: (ii) if species have more restricted distribution, the chance that a researcher can find it is lower than for species with larger geographic ranges. Thus, we measured also the proportion of species new to science for each taxonomic group. The expectation is, again, that organisms with high dispersal potential are less likely to provide new species for science when studying new areas.

One of the expectations of the ubiquity theorem [5] is that, locally, a large representation of the global species pool is present: if species are widely distributed, they also occur (almost) in any place where the environmental features suite them. Thus, we tested this assumption at two spatial scales: (iii) regionally, using as response variable the proportion of species found in the survey, Northern Sardinia or Western Sweden (regional diversity) compared to the total number of species known worldwide for the taxon (global diversity); (iv) locally, using the proportion of species found in each single sample (local diversity) compared to the total number of species in the species pool for each area (regional diversity), identified as the total number of species found in each survey (Northern Sardinia or Western Sweden). To reduce the effect of potential sampling bias for hypotheses (iii) and (iv), we repeated the analyses using estimates of species richness instead of the actual observed richness, using the Chao1 estimator from incidence data [18]. This estimator is able to reliably extrapolate the potential number of additional species than can be found in the area by further sampling, given the actual observed number of species and how many of these have been found only once or twice.

#### Statistical models

Other variables that could influence the results of the statistical analyses, with potential pseudoreplication, are the taxa themselves and the sampling site. To be able to account for a combination of such fixed and random effects in the models, we used Linear Mixed Effect Models (LMEMs) that have been designed exactly for these kinds of analyses, with violations of the assumption that data are independent [19]. Thus, we implemented several models, one for each of the four response variables, each one accounting for a different proxy of diversity, namely (i) proportion of species with restricted distribution, (ii) proportion of new species for science, (iii) proportion of global species pool found regionally and (iv) proportion of regional species pool found locally. Among the explanatory variables, we disregarded the uninformative ones that had no, or almost no variability in the dataset, or that were highly correlated with other, more informative variables: thus, two variables were not included in the models. The proportion of parthenogenetic species was not included because it was correlated with body size: only the groups with the smallest body size, Gastrotrichs and Rotifers, had strictly parthenogenetic species. Depth was not retained because of its low variability among taxonomic groups: all groups had the same depth range, from 0 to about −35 m, and only one sample at −70 m contained acoels and one sample at −50 m contained gastrotrichs. The structure of the model was the same in the four cases, with the ecologically meaningful explanatory variables for each taxonomic group accounting for four fixed effects: body size, proportion of endobenthic species, proportion of species with dispersing stages, and proportion of occupied habitat types. The taxonomic group was included as a random effect; the sampling site, with two levels only (Northern Sardinia or Western Sweden), was included in the model as a fixed effect in order to obtain reliable estimates of variance [7], [20], [21]. All explanatory variables were always retained in each analysis, and no model simplification was performed.

A binomial distribution was assumed in all models, as the response variables are proportion data. Values of Chao estimates of local and global diversity were rounded to the nearest integer to allow the use of binomial distribution in all models.

The significance and importance of each explanatory variable in the models were evaluated using model averaging as described in Burnham and Anderson [8]. First, for each of the four analyses, the full model was generated; then, the set of sub-models including all possible combinations of the explanatory variables was generated, and the relative importance of each variable was calculated, on a scale from 0 to 1, as the sum of the Akaike weights of the sub-models in which the variable appears; better models have larger Akaike weights, and a variable that contributes more to model fit will thus have a higher relative-importance value. Parameter estimates and unconditional standard errors for each explanatory variable were calculated by averaging over all sub-models in which the variable appears, weighting values from individual sub-models by the sub-models' Akaike weights. We will base the significance of the results on the more robust relative-importance values from model averaging, and not on the p-values, more easily affected by the structure of the statistical models; nevertheless, we will report both values and discuss discrepancies, when present.

All analyses were performed with the statistic software R 2.13.2 (R Development Core Team 2011: http://www.R-project.org), LMEMs with package *lme4* 0.999375-39 [22], Chao estimates with package *vegan* 1.15-4 [23], and model averaging with package *MuMIn* 1.6.5 [24].

## Results

### Sardinian fauna and remarks

**Figure 1:**
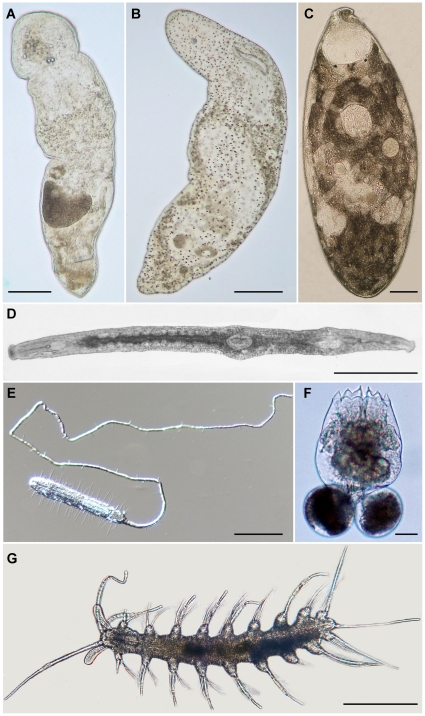
Representatives of the soft-bodied meiofaunal taxa considered in the analyses. A, *Flagellophora* sp. -Nemeretodermatida; B, *Proporus* sp. -Acoela; C, *Polycystis naegelii* -Rhabdocoela: D, *Parotoplana renatae* -Proseriata; E, *Urodasys viviparus* -Gastrotricha; F, *Brachionus ibericus* -Rotifera; *Mesonerilla intermedia* -Annelida. Light microscopy phomicrographs, scale bars A, C, E = 100 µm, B, D, G = 250 µm, F = 20 µm.

Details on the selected soft-bodied meiofaunal groups (Figure 1) from the Northern Sardinia workshop are provided below. The complete list of species found in Northern Sardinia is given in Tables S3, S4, S5, S6, S7, S8.

#### Acoela and Nemertodermatida (Tables S3)

These two taxa, formerly classified within Platyhelminthes, are currently regarded as basal bilaterian clades [25]–[27], or, alternatively, as dramatically reduced deuterostomes [28].

By December 2010, there were published records of 56 species of Acoela from the Mediterranean, 22 of which from Italian waters. In comparison, 57 acoel species were recorded just from the littoral zone of the 99 km^2^ German island Sylt, and 47 species were recorded from the 57 km^2^ Gullmaren fiord on the Swedish west coast (see http://acoela.myspecies.info and UJ unpublished). Clearly acoel diversity in the Mediterranean is understudied, and there is an enormous potential for finding new species. The twenty species of Acoela found during the workshop are, with the exception of two, new to science. The exceptions are *Symsagittifera corsicae* Gschwentner, Baric & Rieger, 2002, whose type locality is at the nearby island of Corsica [29], and *Paratomella rubra* Rieger & Ott, 1971, a potential widely distributed acoel.

Nemertodermatida were revised by Sterrer [30]. The taxonomy of Nemertodermatida is particularly problematic, with broadly defined nominal species of which three were previously reported from the Mediterranean [30]. Of the five species of Nemertodermatida found, only *Nemertinoides elongatus* Riser 1987– reported by Sterrer [30] from the Mediterranean (Rovinj, Croatia) - could be determined with some degree of reliability. The others could only be identified to genus level, pending a revision of the taxon which takes into account molecular data.

The taxonomy of Acoela and Nemertodermatida is plagued by the vague (by modern standards) original descriptions of many taxa. Given the limited amount of morphological diagnostic features, the topic of species delimitation in the two taxa should be readdressed, and, in many instances, recourse to molecular information is deemed fundamental. Therefore, at this time, reports of species outside the type locality should be considered with caution unless corroborated by nucleotide sequences.

#### Platyhelminthes: Proseriata (Table S4)

The composition of Proseriata in the Mediterranean has received particular attention. Fifty-seven proseriate species are currently reported from the northern sector of the central-western area of the Mediterranean, where La Maddalena National park is located [31]. Intensive research in other areas of Sardinia, Corsica, and Tuscany [32] makes the sector among the best studied in the world.

Nonetheless, of the 34 species found, more than 50% (18) are undescribed. Most of the new species belong to the genera *Archimonocelis* Meixner, 1938, *Duplominona* Karling, 1966, and *Parotoplana* Meixner, 1938. Paradoxically, research on these three genera has been particularly intense, even with the production of monographs based on species from the central Mediterranean [33]–[36]. One genus, *Parotoplana*, was particularly well represented in the samples, and most (7 out of 10) of the species found are new. Two of them belong to the complex *Parotoplana renatae/macrostyla*, which includes a number of similar, poorly delimited taxa [28], whose taxonomical resolution would benefit from the contribution of molecular information.

The distribution of most of the known species appears limited to central-western Mediterranean. Indeed, the type locality (and, in some instances, the only locality from which the species was known) of quite a few of them is located within the La Maddalena Archipelago (*Parotoplana geminispina* Delogu & Curini-Galletti, 2009) or in nearby Corsica (*Nematoplana corsicana* Curini-Galletti & Martens, 1992, *Archimonocelis staresoi* Martens & Curini-Galletti, 1993, *A. meixneri* Martens & Curini-Galletti, 1993, *Duplominona corsicana* Martens, 1984, *D. longicirrus* Martens, 1984) [33], [34], [37], [38].

Two of the species of uncertain taxonomic attribution (*i.e. Coelogynopora* cf *gynocotyla* Steinböck, 1924 and *Monotoplana* cf *diorchis* Meixner, 1938) have a range that encompasses the Atlantic coasts of Europe [39], [40]. However, specimens of *M.* cf. *diorchis* from the Mediterranean differ in chromosome number [4] from populations from northern Atlantic, where the type-locality (Kieler Bucht) is placed [41]. *C. gynocotyla* is the only *Coelogynopora* Steinböck, 1924 without copulatory and/or accessory sclerotised structures, and therefore lacks one of the basic information for species discrimination. A thorough revision of the two taxa, with the inclusion of molecular data, is therefore needed before any decision of the status of the populations from La Maddalena can be attained.

*Boreocelis* cf. *urodasyoides* Ax, 1963 is tentatively attributed to a species whose original description lacks crucial details on the morphology of the sclerotised structures and should be implemented with more information on specimens from the type locality (gulf of Naples) [41]. One single specimen of *Philosyrtis* sp. was found, in bad state of preservation, making identification impossible.

Overall, data confirm the incomplete state of knowledge of the Proseriata even in one of the most studied areas of the Mediterranean. Furthermore, the finding of numerous species whose distribution appears limited to the northern sector of the central-western area suggests a high level of endemism of proseriate taxa.

#### Platyhelminthes: Rhabdocoela (Table S5)

Rhabdocoela is a very species-rich taxon of rhabditophoran flatworms, which can be recognised by a true pharynx bulbosus and a specific construction of the protonephridial flame cell [13], [42]. Worldwide about 1550 species are described, 60 percent of which (about 930 species) are from marine or brackish water. One hundred and seventy nine marine rhabdocoel species are known from the Mediterranean, 97 of which are Mediterranean endemics. Not included in these counts are the 17 species known only from the Black Sea. Of the 179 Mediterranean species, 146 occur in the Western Mediterranean, as defined by Spalding et al. [43]. Of these 146 species, 75 are endemic for the Western Mediterranean, at least as far as is known at present. The relative high number of species known from the Western Mediterranean as compared to the rest of the Mediterranean is clearly because of sampling bias, as the coastal area of Marseille was intensively sampled by Michel Brunet in the sixties and seventies of the former century, and many species, albeit only kalyptorhynchs, were described by him in a series of papers [44]–[54]. Moreover, a large study was published by Ax [55], in which he described several species from coastal salt marshes between Narbonne and Perpignan. In literature, only three species are mentioned from Sardinia: *Trigonostomum penicillatum* (Schmidt, 1857), *T. venenosum* (Uljanin, 1870) and *Djeziraia euxinica* (Mack-Fira, 1972) [14], [56].

In the material collected during the workshop at La Maddalena, 54 species of rhabdocoels were collected. Thirty-two of these species belong to the Kalyptorhynchia, 22 to its sister taxon Dalytyphloplanida (for a taxonomical overview of the taxon Rhabdocoela see Willems et al. [13]). Additionally, an unknown member of (probably) the genus *Ciliopharyngiella* Ax, 1952, a taxon of uncertain affinities, was also found. This species is mentioned in Tables 1 and S5, but not further considered in the following text. Only 20 of the species found could be identified as already described. Thirteen species were represented by juveniles, or by specimens that do not allow identification. It is therefore likely that the number of species new to science found (21) is to be considered as a conservative estimate.

**Table 1:** Number of species found in Northern Sardinia and in Western Sweden for each taxon.

	No. species found	Undescribed species	Uncertain status
**Northern Sardinia**			
**Taxon**			
Acoela	23	21	0
Nemertodermatida	5	0	4
Proseriata	34	18	1
Rhabdocoela	55	21	13
Gastrotricha	60	17	6
Annelida*	13	2	4
Rotifera	16	0	5
TOTAL N Sardinia	203	76	33
**Western Sweden**			
**Taxon**			
Acoela	21**	6**	0
Nemertodermatida	6	2	0
Proseriata	21	3	0
Rhabdocoela	35	3	1
Gastrotricha	43**	11	0
Annelida*	6	0	0
Rotifera	23	0	2
TOTAL W Sweden	154	25	3

* Include only records from exclusively endobenthic families.

** The original estimate reported by Willems et al [4] were lower. The current numbers are the result of subsequent taxonomic studies on additional material.

Several of the known species found have a wide distribution across the Mediterranean and the Atlantic coasts of Europe, the most-studied areas in the world for turbellaria. However, one of these wide-ranged species, *Gyratrix hermaphroditus* Ehrenberg, 1831, is a notorious example of cryptic diversity, probably containing a large amount of separate sibling species [57]. A detailed knowledge of the composition and range of the siblings is still lacking, and the specimens found at la Maddalena can only be reliably identified in a molecular revision of the group, which is presently under way.

Five of the known species are Western Mediterranean endemics: *Austrorhynchus bruneti* Karling, 1977, *A. karlingi* Brunet, 1965, *Carcharodorhynchus multidentatus* Brunet, 1979, *Duplacrorhynchus megalophallus* Artois & Schockaert, 1999 and *Rogneda colpaerti* Artois, 2008, while six are recorded for the first time for the Mediterranean proper (excl. the Sea of Marmara). Four of these six were previously only found in the Black Sea and the Sea of Marmara, and presumably have a more widespread, circum-Mediterranean distribution: *Baltoplana valkanovi* Ax, 1959, *Progyrator mamertinus* Graff (1874) Reisinger, 1926, *Promesostoma ensifer* (Uljanin, 1870) Pereyaslawsewa, 1892 and *Promesostoma maculosum* Ax, 1956. *Cystiplana paradoxa* Ax, 1954 was previously found in the Black Sea, the Sea of Marmara and the island of Sylt (European N. Atlantic), and probably has an even wider distribution. The sixth species new to the Mediterranean, *Trigonostomum australis* Willems et al, 2004, was up to now only found along the Australian East Coast [56] and therefore apparently has an extreme disjunct distribution. However, the worldwide distribution of microturbellaria is very poorly known, and it could well be that the species is much more widespread. On the other hand, it could also be that the populations from the Mediterranean and Australia will appear to be genetically separated, and actually represent cryptic species. A similar case is the finding of *Gyratrix proaviformis* Schockaert & Karling, 1977, a species hitherto only known from the Pacific coast of the US (Oregon), in Punta Negra (Sardinia) in March 2010 (B. S. Tessens & W. R. Willems, pers. comm.). Only a thorough broad scale sampling and the use of molecular techniques can help to solve these intriguing cases of widely separated, apparently conspecific, populations, which are illustrative of our lack of knowledge as it comes to biogeography and biodiversity of microturbellaria.

#### Gastrotricha (Table S6)

The phylum Gastrotricha is cosmopolitan with approximately 780 species divided into two orders: Macrodasyida, with about 324 strap-shaped species, all but two of which are marine or estuarine and Chaetonotida with about 455 tenpin-shaped species, over 30% of which occur in salty environments [58], [59]–[63]. The Italian marine gastrotrich fauna has been the focus of much research and numerous sampling campaigns, and, with approximately 180 species recorded in more than 230 localities [64]–[66], ranks among the best known in the world. Indeed, among meiobenthic phyla, none enjoys such a detailed knowledge of species composition and distribution around the Italian coasts. Yet, of the 60 species of the phylum found during the workshop, 17 species are still undescribed. This number includes also species found previously in the Mediterranean, awaiting formal description, and does not entirely reflect species unique to La Maddalena area. However, the discovery of two new species, belonging to two undescribed genera that could not be easily placed into any extant family, has been surprising, and witnesses the incomplete state of knowledge in the Mediterranean even of the Gastrotricha. Both these species were found in samples collected into marine caves, which are confirmed as hot-spots of diversity for the group [67], [68].

From a biogeographical point of view, most species appear to have a wide distribution across the Mediterranean [69] and the Atlantic coasts of Europe with some known to be regional cosmopolitans (e.g. Macrodasyida: *Acanthodasys aculeatus* Remane, 1927, *Dactylopodola typhle* (Remane, 1927), *Urodasys viviparus* Wilke, 1954 etc.; Chaetonotida: *Aspidiophorus paramediterraneus* Hummon, 1974, *Heteroxenotrichula pygmaea* (Remane, 1934) etc.). Two species are reported here for the second time along the Italian coasts: one species, *Diplodasys sanctaemariae* Hummon & Todaro, 2009, originally described from Apulia but known also from the Levantine Basin [65]; the other, *Tetranchyroderma aapton* Dal Zotto, Ghiviriga & Todaro, 2010, recently described from Capo Caccia [66], a Sardinian locality not too far from the current sampling area; however, at Costa Paradiso the species appears most abundant. Of particular interest is the finding of *Acanthodasys* cf *caribbeanensis* Hochberg & Atherton, 2010, which constitutes the first report of the species for the Mediterranean [70]. The taxonomic status of the population found at La Maddalena, will however be assessed on comparison with the Caribbean worms on the basis of molecular genetics.

At higher taxonomic level it may be noticed that while the highly diversified Thaumastodermatidae [71] is well represented in our samples, it is not so for the second- and third-most speciose marine gastrotrich families, as Turbanellidae and Macrodasyidae are present at La Maddalena with only five species each. Within these taxa the genus *Macrodasys* Remane, 1924 (Macrodasyidae) is especially under-represented whilst *Turbanella* Schultze, 1853 (Turbanellidae) is absent altogether.

Marine gastrotrichs are strictly interstitial organisms (with few exceptions), consequently our sampling efforts usually focus on clean sandy sediments collected from very shallow areas (1–3 m water depth); the relevance of the abundant and diverse fauna yielded by some of the sediments collected during the work-shop held at La Maddalena strongly calls for widening the surveys to deeper sediments usually neglected in gastrotrich faunistic investigations.

#### Annelida (Table S7)

Annelida contains more than 17000 species worldwide, widely spread among marine, limnic and terrestrial environments [72], [73]. Most marine representatives of the group belong to the macrofauna, and their composition and distribution along the Italian coasts is considered to be adequately known [74]. Meiofaunal taxa belong to several, unrelated groups [75], and have been studied far less [74].

This investigation focused on exclusively interstitial families, and did not comprise the interstitial representatives of macrofaunal families. Members of Nerillidae, Protodrilidae, Psammodrilidae and Polygordiidae were recorded, with a total of thirteen species. Seven species were collected at the coastal stations at La Maddalena and Costa Paradiso and eight at Capo Caccia (Nereo cave). Only four of these are previously reported from the Mediterranean Sea [74], indicating a hitherto unseen diversity of both known and unknown meiofaunal Annelida in the Mediterranean.

Among Nerillidae, *Nerillidium mediterraeum* Remane, 1928 and *Mesonerilla intermedia* Wilke, 1953 were the most abundant taxa. Both species have been reported previously from several European locations in the Atlantic and Mediterranean. In the present study *Nerillidium mediterraneum* was collected at six stations, *M. intermedia* at five, and *M. armoricana* Swedmark, 1959 and *M. biantennata* Jouin, 1963 at two. The most remarkable findings occurred in the Nereo Cave, which showed a great diversity of nerillids. These included a new species of *Mesonerilla* with long palps and pygidial cirri. *Mesonerilla* spp. have previously been recorded in other cave systems of the Atlantic [15], [76] as well as in Pacific hydrothermal deep sea vent areas [77]. Taking into account that this genus is pending revision [78] and may turn out paraphyletic, it still seems highly plastic and suggesting an interesting zoogeographical history. Single juvenile specimens of *Meganerilla* Boaden, 1961 and *Trochonerilla* Tzetlin and Saphonov 1992 were also recorded at the Nereo cave. *Meganerilla* sp. resembles the North Atlantic *M. swedmarki* Boaden, 1961 by the lack of median antenna. *Trochonerilla* is so far monospecific, however, further material is necessary to determine the species status. It is noteworthy that this is the first finding of *Trochonerilla* outside tropical aquariums (including the type locality of the Moscow Aquarium). This Mediterranean finding therefore most likely represents a new species, geographically distant from the presumed natural, far-east, tropical habitat of *T. mobilis* Tzetlin and Saphonov, 1992.

Several specimens belonging to *Polygordius* were also found in the Nereo cave gravel sediments, which is the first record of Polygordiidae from a cave. The lack of specimens with the pygidium intact prevented further identification, however, several species of *Polygordius* are previously reported from the Mediterranean, including Italy [79].

Three species of Protodrilidae were found, always with low abundances: *P. gracilis* Von Nordheim, 1989, *P. similis* Jouin, 1970 and *P. purpureus* (Schneider, 1868). *Protodrilus gracilis* was recorded at three stations at La Maddalena, always in coarse sandy environments. This species was previously reported from the Mediterranean bay of Naples (Italy) and Banyuls-sur-Mer (France), as well as from the Atlantic coasts of northern Europe [80]. *Protodrilus similis*, from intertidal fine sandy sediments at Punta Rossa, was previously reported from southern Mediterranean at Gulf of Tunis (Tunisia) and the Atlantic at Archachon bay (France). *Protodrilus purpureus* is here recorded for the first time from marine caves, however several Mediterranean records exist [81].

*Psammodrilus* from Cavaliere bay represents the second record of the genus in the Mediterranean besides *P. balanoglossoides* Swedmark, 1952 [81]. *Psammodrilus* sp. differs significantly from the similarly small sized Atlantic European *P. fauveli* (Swedmark, 1958), but is surprisingly similar in both morphology and preliminary DNA comparisons to the recently described West Atlantic, Bermudian, *P. moebjergi* Worsaae and Sterrer, 2006.

#### Rotifera (Table S8)

Rotifera are group of microscopic aquatic animals with about 2000 described species. Most rotifers live in freshwater and limno-terrestrial habitats, and only about 400 species have been found in saline waters so far [16], [82], [83]. Surprisingly, very few taxonomic and faunistic studies have dealt with marine rotifers, and most of the present knowledge on rotifer distribution is limited to the freshwater habitat.

The geographical distribution of the brackish and marine rotifers (as well as that of most freshwater ones) largely reflects the distribution of rotifer investigators [84], [85], consequently limiting biogeographical comparisons. Nevertheless, the Mediterranean is one of the best known areas in the world, but few specific investigations have been carried out in Italy [86]. Almost every new study dealing with marine rotifers from the Mediterranean and Italian coasts is likely to reveal new species to the area or to science [87], [88].

The habitats we sampled in Northern Sardinia (Table S1) provided 16 species of rotifers based on morphological criteria. Five of them were identified to genus level only, and can be potential new species to the area or to science. Among the other 11 species, two are new for the Italian marine fauna, but were already known from the Mediterranean (Table S8).

Rotifers notoriously host a large hidden diversity, with several cryptic species for many morphospecies [89]. Thus, we used DNA taxonomy to identify some of them and to look for potential cryptic taxa, by amplifying and sequencing a fragment of the cytochrome c oxidase subunit I (COI) from few individuals of each sample.

The species complex *Brachionus plicatilis* Müller, 1786 is present in the Mediterranean and it may be difficult and ambiguous if not impossible to identify some of its cryptic taxa from morphology only. DNA taxonomy identified one of our samples as belonging to *B. ibericus* Ciros-Peréz, Gómez & Serra, 2001 (Table S8, GenBank accession numbers HQ444171-HQ444172), a member of the group of small-sized species in the complex. Uncorrected genetic distances between our sequences and the ones available in Genbank ranged from 0.0 to 3.7%. This species has never been found in Italy before, and was known only from Spain, Greece and the United Kingdom so far [90]–[92].

Both *Brachionus urceolaris* Müller, 1773 and *Lecane bulla* (Gosse, 1851) are euryhaline species [82], and the COI sequences of both confirmed that our marine samples belong to these morphospecies (GenBank accession numbers for *B urceolaris*: HQ444169–HQ444170; for *L. bulla*: HQ444174). Nevertheless, DNA taxonomy suggested also that our marine populations may be cryptic taxa different from the ones previously sequenced from freshwater habitats, as uncorrected genetic distances between the marine and freshwater ones are comparable to the distances between cryptic taxa of other rotifer species complexes that have been tested for reproductive incompatibility [93], [94]. Distances were between 18 and 21% for *B. urceolaris* [95]–[97] and between 8 and 17% for *L. bulla* [96], [98], [99].

The sequences we obtained for *Testudinella clypeata* (Müller, 1786) are the first ones available (GenBank accession numbers HQ444166–HQ444168), so we cannot test its identification using DNA taxonomy. All individuals we found were morphologically homogeneous; nevertheless, we could identify two cryptic species, with genetic distances of 20%. This is quite a high distance, as the distance between the latter two cryptic species and the only other species with available COI sequences, *T. patina* (Hermann, 1783), is 28%.

### Overview of the two workshops

A summary of the faunistic results from both sampling campaigns in Northern Sardinia and in Western Sweden is given in Table 1. The complete list of species found in Western Sweden is reported in Table S2; for general comments on these taxa refer to [4].

In general, soft-bodied meiofauna is richer in Northern Sardinia than in Western Sweden (203 vs 154 species); this is particularly true for strictly interstitial taxa such as Gastrotricha (60 vs 43 spp); on the other hand, taxa known to prefer fresh- or brackish waters, such as rotifers, are less abundant in the Mediterranean samples (16 vs 23 spp). Acoela and Nemertodermatida are present in the two areas with a vey similar number of species (23 vs 21 spp and 5 vs 6 spp respectively). Over 37% of the species found in Sardinia appear to be undescribed taxa, although half of them require additional studies; in contrast to the status of only 16% of the species found in Sweden considered as undescribed.

### Correlates of biological diversity

The potential explanatory variables included in the models, namely body size, proportion of endobenthic species, proportion of species with potential for dispersal, and proportion of occupied habitat types, controlling for sampling site and taxonomic group, provided evidence of significant global forces driving patterns of diversity, acting in the same way in different geographical areas. The statistical models significantly explained variance in the four descriptors of diversity we used as response variables (Table 2), namely proportion of species with restricted geographic range, proportion of new species, regional to global proportion and local to regional proportion.

**Table 2:** Model-averaged parameter estimates.

	(i)			(ii)		
	Restricted distribution			New species		
	Estimate			Estimate		
	± SE	RI	p	± SE	RI	p
(Intercept)	−0.44±0.43	-	0.310	−0.71±0.64	-	0.266
Body size	0.11±0.10	0.09	0.264	−0.19±0.12	0.21	0.067
Dispersal	**−1.71±0.54**	**0.96**	**0.001**	**−3.78±1.60**	**0.97**	**0.018**
Habitat types	0.32±0.96	0.13	0.739	−0.49±1.89	0.12	0.791
Endobenthic	0.58±0.63	0.09	0.357	−0.75±0.99	0.05	0.450
Sampling site	−0.46±0.25	0.49	0.041	**−0.96±0.34**	**0.97**	**0.004**

	(iii)			(iii a)		
	Regional/global			Regional/global (Chao)		
	Estimate			Estimate		
	± SE	RI	p	± SE	RI	p
(Intercept)	−3.28±0.42	-	<0.001	−0.44±0.43	-	0.001
Body size	−0.19±0.12	0.21	0.106	**−0.91±0.13**	**1.00**	**<0.001**
Dispersal	−0.28±0.55	0.07	0.615	1.82±1.14	0.07	0.112
Habitat types	0.95±0.33	0.84	0.003	0.30±0.20	0.10	0.130
Endobenthic	−0.07±0.51	0.05	0.885	**1.84±0.59**	**0.94**	**0.002**
Sampling site	−016±0.24	0.18	0.510	N/A	0.00	N/A

	(iv)			(iv a)		
	Local/regional			Local/regional (Chao)		
	Estimate			Estimate		
	± SE	RI	p	± SE	RI	p
(Intercept)	−1.92±0.32	-	<0.001	−3.14±0.72	-	<0.001
Body size	0.09±0.15	0.11	0.523	0.39±0.17	0.44	0.027
Dispersal	0.50±0.50	0.17	0.315	1.21±0.54	0.49	0.027
Habitat types	−0.21±0.84	0.09	0.803	0.74±1.40	0.17	0.598
Endobenthic	0.16±0.63	0.09	0.795	−0.16±1.03	0.07	0.875
Sampling site	−0.35±0.43	0.17	0.310	−0.65±0.34	0.33	0.0061

Relative-importance values (RI) and p-values for the six models with all ecologically relevant variables retained in the models. Identification codes from (i) to (iv) refer to the four models explained in the text; codes followed by ‘a’ refer to analyses using Chao estimates of regional diversity. Parameters with high relative-importance values are highlighted in bold.

The proportion of species with restricted distribution in each taxonomic group ranged from 0 to 95% and was significantly negatively related to the proportion of species with dispersal abilities (Table 2-i): taxonomic groups with more species able to disperse (e.g. rotifers and annelids) are the groups where less species are restricted in their distribution and where more species have wide distributions. The proportion of species with restricted distribution was overall higher, but only marginally significantly, in Sardinia (69%) than in Sweden (41%) (Table 2-i); moreover, the low relative-importance value for this predictor demonstrates a low effect of the differences between Northern Sardinia and western Sweden on the patterns of distribution of meiofauna.

The proportion of species new to science ranged from 0 to 90% in different taxonomic groups and showed a similar scenario, negatively related to the proportion of species with dispersal abilities (Table 2-ii): the probability of finding new species in new surveys is higher in taxonomic groups where dispersing stages are not present, e.g. acoels, proseriates and gastrotrichs. The proportion of new species for science for the soft-bodied meiofauna was significantly higher in Sardinia than in Sweden (Table 2-ii), especially for taxa such as Acoela (91% vs 29%), Proseriata (55% vs 14%) and Rhabdocoela (50% vs 9%).

The proportion of the global species pool represented at the regional scale showed significant patterns with high relative-importance values only when accounting for potential sampling bias by using Chao estimates instead of the actual observed number of species (Table 2-iii, iii a). Using Chao estimates as descriptors of regional species diversity, body size had a negative influence, whereas the proportion of species that are exclusively endobenthic had a positive influence. The regional representation of the global species pool is higher in groups with small body size and mostly living as endobenthic.

The proportion of the regional species pool found in each single sample showed significant patterns related to body size and dispersal abilities only when accounting for potential sampling bias by using Chao estimates for regional richness (Table 2-iv, iv a), even if none of the variables had high relative-importance values as predictors of the model.

## Discussion

Two main results were produced by the detailed taxonomic surveys in Northern Sardinia and in Western Sweden. First, the number of new, still undescribed species is high even in well-studied areas; second, the patterns of diversity in meiofauna have strong macroecological correlates, such as body size, dispersal ability and occurrence in endobenthic habitat. As hypothesised, these macroecological correlates overcome the effect of local variables; the only significant differences in the patterns of diversity between Northern Sardinia and Western Sweden refer to the amount of undescribed diversity. This issue deserves additional explanations, which we provide in the following paragraph.

### Undescribed diversity

Of the 203 species found in Northern Sardinia, 76 (about 37% of the total) have been recognized as previously undescribed by authorities in their field. As impressive as the percentage may be, it may turn out to be a conservative estimate. In fact, a relatively high, additional number of species (33) could not be identified with certainty, due to the fact that the material was inadequate, or the specimens found belonged to groups where revisions are pending, and a portion of them may turn out to be new species as well. In comparison, during the workshop held at Tjärnö in 2007, for the same taxa considered here, 143 species were found, only 13 of which were new to science. The Tjärnö workshop spanned a longer time, and the sampling effort was remarkably more intense: during a two week period, almost 100 samples were sorted, from littoral beaches, rock pools and different types of sublittoral sand and mudflats, to a depth of about 90 m on *Lophelia* reefs [4]. Furthermore, the workshop hosted a greater number of researchers, who worked on different sediments and habitats at the same time, sharing findings among each other. Yet, the number of new species in Northern Sardinia is much higher, both in absolute and in relative terms.

Differences in both the total number of species and in the proportion of undescribed species found during the workshops are remarkable. These differences may reflect reality or may be artefacts of sampling effort. The effect of sampling bias and sampling effort is a known problem in all biodiversity inventories, even in well-known organisms such as birds and ground beetles [100], [101], but becomes massive in inconspicuous meiofaunal organisms [102]. Interestingly, the proportion of undescribed diversity was different between the two workshops only for acoels, proseriates and rhabdocoels, and higher in Sardinia. Given that Sweden has a long history of taxonomy on acoels and flatworms [103]–[105], it is not surprising that most of the species in these three groups have already been discovered around the island of Tjärnö.

Most of the subtidal samples examined during the workshop held at Tjärnö were taken by a dredge or a Warén sledge [4], the samples were later stored in large plastic boxes in a cold room, in order to allow the animals to crawl to the surface of the sediment. The whole process, however, may have proved noxious to minute and fragile organisms. On the contrary, the sediments studied in La Maddalena were carefully and manually collected by scuba-divers from the oxygenated surface layers only, in order to minimize any damage to interstitial organisms. Microhabitats representing the greatest diversity of sediment could therefore also be selectively chosen by the divers, and intermixing of habitats avoided. This method is also far more efficient for extracting animals limited to a concentrated surface layer and allows for immediate processing of the sediment yielding a more vivid and possibly richer fauna. Moreover, there may be a role of previous knowledge in the area: the census of marine fauna at Tjärnö is undoubtedly more complete than in any site of the Mediterranean. Tjärnö lies in an intensely studied area, with the presence of numerous, closely located Marine Biological Stations (to quote the most famous, Helsingør (Denmark), Sylt (Germany); Sven Loven Centres at Kristineberg and Tjärnö itself (Sweden). On the contrary, Marine Biological Stations in the Mediterranean are far fewer and farther apart – in the case of La Maddalena, the workshop was hosted in three adjacent rented flats, and microscopes were personally carried by the researchers involved. The lack of suitable, coastal locations where samples can be processed affects particularly the knowledge of soft-bodied meiofauna, which needs to be studied alive. Nevertheless, the number of species in Northern Sardinia was higher than in Western Sweden.

Differences between the two surveys are indeed present: in the kind of habitat (mostly silt and mud in Sweden, and clean sand in Sardinia), in sampling techniques (mostly related to the differences in the sediments themselves), in the climate, in the latitudinal position and in the biogeographical area (see discussion in the last section). Notwithstanding these obvious differences, our macroecological analyses revealed common patterns of diversity correlating with the same variables in the two areas: the number of new, undescribed species with restricted distribution is higher in taxonomic groups with no dispersing stage regardless of size of the organisms. The fact that size of the organisms did not correlate with the number of new species per group is in contrast to the ubiquity hypothesis. According to this paradigm, smaller organisms should have wider geographical ranges, and thus, the probability of finding new species in local samples should be negatively correlated to body size [5]. Body size in the meiofaunal organisms analysed in Northern Sardinia and Western Sweden ranged from 0.08 mm to 13 mm, encompassing three orders of magnitude. Thus, its absence from the important explanatory variables is not due to lack of variability, but to an actual pattern: the absence of dispersing stage but not body size influences the probability of finding new species with restricted distribution in meiofauna. Thus, further studies aimed at describing diversity in marine meiofauna should focus primarily on such organisms in order to provide new data for the accurate description of marine diversity.

### Patterns of diversity in space

The presence of dispersing ability, body size, and the endobenthic habitat where the organisms live are significant correlates of species distribution in space at different scales. Dispersal abilities influence the patterns of distribution, as expected from the ubiquity hypothesis [5], [6]; this result is robust and consistent, given the high relative-importance values in the models (Table 2-i, ii) and the fact that such capability is an important predictor for both the proportion of species with restricted distribution and for the proportion of species new to science. On the other hand, small, strictly endobenthic species, both in Northern Sardinia and in Western Sweden, have a high representation of the global species pool at the regional scale. The fact that small organisms have a high regional to global proportion is in accordance with the ubiquity hypothesis [5], [6]: our results support the scenario that, if organisms are small, most the available global species pool will be found sampling different habitats at a regional scale. At the largest scale, that is comparing Northern Sardinia and Western Sweden (regional diversity) with the overall worldwide diversity (global species pool) of each meiofaunal group, a larger representation of the global species pool is present in smaller meiofaunal groups, as expected from the ubiquity hypothesis [102]. Still this significant correlation could be due to taxonomic bias, with a better taxonomic resolution in larger organisms, and a higher degree of hidden diversity in smaller than in larger meiofaunal organisms [89], [106], [107].

The relative influence of body size in structuring diversity in space changes at different spatial scales. At the local spatial scale, the number of species found in each sample in proportion to the potentially available ones for each of the two areas (regional species pool) is not related to any of the analysed predictor, not even to body size or to the endobenthic habit. Thus, body size, negatively related to spatial distribution at the regional to global scale, becomes non-influential at the local to regional scale. This fact could be explained by the following scenarios: meiofaunal groups with larger body size can move freely at the local to regional spatial scale, at least as much as the ones with smaller body size. On the other hand body size may become a limiting factor to dispersal from the regional to global spatial scale. This pattern is consistent between Northern Sardinia and Western Sweden, with no differences between the two areas.

### Differences between Northern Sardinia and Western Sweden

Whilst similarities exist in the diversity patterns in meiofauna in these two areas, several differences are indeed present. Other than sampling effort and potential bias in taxonomic knowledge already discussed, there are differences in the kind of habitat. Most of sediments collected in Northern Sardinia ranged from clean, fine to coarse sand, to shelly gravel, including marine caves. This type of sediment favours taxa such as Proseriata [108], Gastrotricha [12], Annelida [75] and Acoela [4]. Conversely, most of the sediments collected at Tjärnö were much siltier, consisting, in many cases, of muddy sand [4]. Water salinity is also different between the two investigated areas and may account for the recorded faunistic differences. Low salinity values and ample variation of this physicochemical factor are known to have an adverse impact on meiofauna biodiversity [2]. The salinity at the littoral and shallow sublittoral stations of Tjärnö may vary from 10 to 34‰ over the year while in Sardinia it is about 38‰ the year around, with little difference between the littoral and the sublittoral sites. Overall species richness in soft-bodied meiofauna undeniably was higher in Sardinia, but we cannot infer whether this could be due to the effect of salinity or to the effect of different species pools in different biogeographical area at different latitudes.

Latitudinal gradients in diversity indeed exist for most organisms: diversity gradients, peaking in the tropics and tailing off toward the poles, are well known biological phenomena, and are shared by both marine and terrestrial systems [109]. Latitude is merely a description of location; nevertheless, it often correlates with other variables that are biologically relevant [110], such as: i) historical events, i.e. the destructive effect of glaciations acting at high latitudes [111]; ii) Rapoport's rule, which attributes the gradient to a decrease in species' ranges toward low latitudes [112]; and iii) differential solar energy input and water availability, linked to biodiversity through productivity [109], [113]. The combined actions of the three factors above and of salinity cannot be ruled out: Sea Surface Temperatures (SST) are indeed markedly different between the two sites. Tjärnö, latitude 58°52′29.12″N, has average offshore SST included between the isotherms of 9°C and 10°C; La Maddalena town (latitude 41°12′45.94″N) has average SST included between 17°C and 18°C (NOAA, National Oceanographic Data Center: www.nodc.noaa.gov). Furthermore, it has been shown that some organisms of the meiofauna may have recolonized the Northern Atlantic from southern refugia [114], witnessing the action of glaciations on boreal marine biodiversity. Finally, the finding at La Maddalena of species only known from neighbouring areas (in cases, with ranges apparently limited to the Corsican-Sardinian complex), also hints that a greater percentage of narrow-range endemics in the Mediterranean cannot be ruled out.

However, the latitudinal influence on diversity of microscopic organisms as meiofauna is still highly debated [102], [115], [116]. Moreover, no differences in the macroecological correlates of diversity could be observed between Sardinia and Sweden, even if there are differences in habitat heterogeneity, and there is a general shortage of suitable well sorted, coarse, and possibly calcareous sediments in the North Atlantic. Poorly sorted sediments provide less pore volume and consequently a low potential for the presence of interstitial meiofauna [2], [108]. Moreover, the fact that the sediment type may be far more restrictive than latitude for meiofauna is supported by several examples of extremely diverse meiofauna in the North Atlantic found in e.g., shell gravel of the Faroe Bank [117], [118], and the coarse sand of Flakkerhuk, West Greenland [77], [119]–[121].

In conclusion, the workshops held at La Maddalena and at Tjärnö, in addition to the wealth of new species found, which will be independently described by the researchers involved, highlighted the very limited knowledge of soft-bodied meiofauna, even in well-studied areas. This result has an impact on the evaluation of the magnitude of the contribution of meiofauna to marine biodiversity, surely underestimated with so many temperate to tropical areas of the planet poorly studied [122] (see also http://coml.org/about).

## Supporting Information

Figure S1 **Sampling localities in Northern Sardinia.**
(TIF)

Table S1 **Detailed information on sampling localities in Northern Sardinia.**
(DOC)

Table S2 **Ecological and biological attributes (adult body length, distribution, endobenthic habitat) for each species included in the analyses.**
(DOC)

Tables S3 **Acoela and Nemertodermatida. Species list and occurrence in Northern Sardinia.**
(DOC)

Tables S4 **Proseriata. Species list and occurrence in Northern Sardinia.**
(DOC)

Tables S5 **Rhabdocoela. Species list and occurrence in Northern Sardinia.**
(DOC)

Tables S6 **Gastrotricha. Species list and occurrence in Northern Sardinia.**
(DOC)

Tables S7 **Annelida. Species list and occurrence in Northern Sardinia.**
(DOC)

Tables S8 **Rotifera. Species list and occurrence in Northern Sardinia.**
(DOC)
