# Supplementary figures and images for: Patterns of Diversity in Soft-Bodied Meiofauna: Dispersal Ability and Body Size Matter
Source: PLoS One. 2012 Mar 23;7(3):e33801. doi: 10.1371/journal.pone.0033801 (PMC3311549; doi:10.1371/journal.pone.0033801)

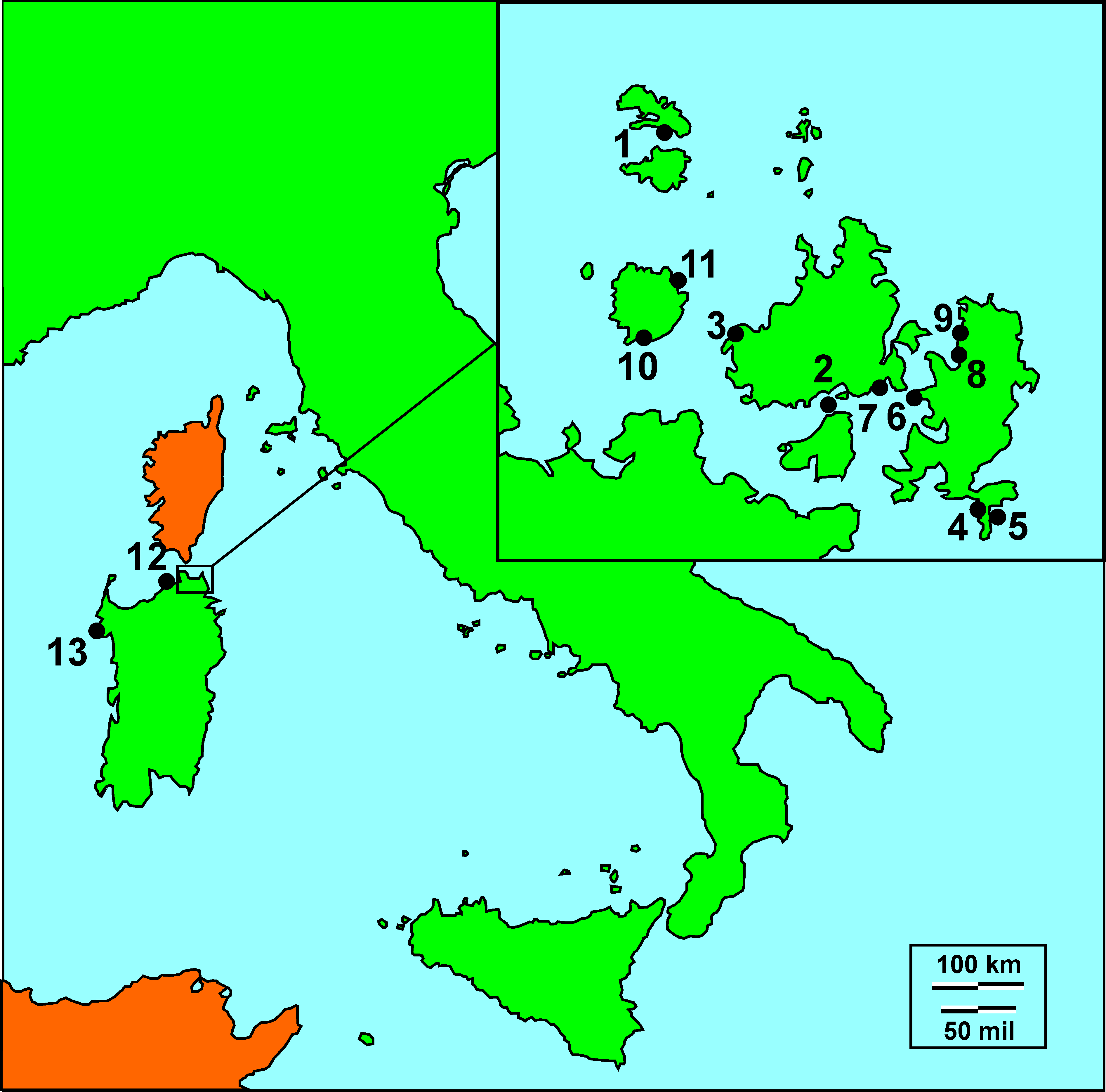

Supplement: Figure S1 — Sampling localities in Northern Sardinia. (TIF) [file pone.0033801.s001.tif]
