# Supplementary material for: Patterns of Diversity in Soft-Bodied Meiofauna: Dispersal Ability and Body Size Matter
Source: PLoS One. 2012 Mar 23;7(3):e33801. doi: 10.1371/journal.pone.0033801 (PMC3311549; doi:10.1371/journal.pone.0033801)
Supplement: Table S1 — Detailed information on sampling localities in Northern Sardinia. (DOC) [file pone.0033801.s002.doc]

Table S1. Detailed information on sampling localities in Northern Sardinia.

| No. | Toponym | Coordinates | Habitat | Date |
| --- | --- | --- | --- | --- |
| 1 | Il Cavaliere | 41°17'29.76''N; 9°20'52.04''E | Medium-fine sand, -33/-37 m | 07/09/2010 |
| 2 | Santo Stefano (Chan) | 41°12'29.22''N; 9°25'48.10''E | Very silty coarse sand, - 6 m | 07/09/2010 |
| 3 | Cala Carlotto | 41°13'44.56''N; 9°22'31.44''E | Medium sand, - 8 m | 09/09/2010 |
| 4a | Punta Rossa West | 41°10'37.3''N; 9°28'10.9''E | Very coarse sand, at ground water level | 09/09/2010 |
| 4b | Punta Rossa West | 41°10'37.3''N; 9°28'10.9''E | Medium to fine sand, intertidal to -3 m | 09/09/2010 |
| 5a | Punta Rossa East | 41°10'36.43''N; 9°28'15.04''E | Medium to fine sand, intertidal to -3 m | 09/09/2010 |
| 5b | Punta Rossa East | 41°10'36.43''N; 9°28'15.04''E | Algae at about - 2 m | 09/09/2010 |
| 6a | Caprera Is. (bridge) | 41°12'53.9''N; 9°26'44.09''E | Sand and algae at about - 0.5 m | 09/09/2010 |
| 6b | Caprera Is. (bridge) | 41°12'53.8''N; 9°26'50.21''E | Sand and algae at about - 0.5 m | 09/09/2010 |
| 7 | Chiesa Is. | 41°12'51.10''N; 9.25'8.94''E | Floating algae at - 0.2 m | 09/09/2010 |
| 8 | Cala Garibaldi | 41°13'19.81''N; 9°27'26.61''E | Sand and algae at - 0.5 m, 38‰ | 10/09/2010 |
| 9a | Cala Serena | 41°13'34.50''N; 9°27'35.62''E | Medium sand, intertidal to -3 m | 12/09/2010 |
| 9b | Cala Serena | 41°13'37.14''N; 9°27'37.38''E | retrodunal pond; salinity: 35‰. Algae at -0.5 m | 12/09/2010 |
| 10 | Cala Soraya | 41°13'51.52''N; 9°21'2.41'' E | medium-fine sand, -2/-3 m | 13/09/2010 |
| 11a | Cala Ferrigno | 41°14'40.38''N; 9°21'23.61''E | Algae fom jetty, - 1.5 m | 13/09/2010 |
| 11b | Cala Ferrigno | 41°14'40.38''N; 9°21'23.61''E | Coarse sand, -4 m | 13/09/2010 |
| 12a | Costa Paradiso | 41°3'8.84''N; 8°56'15.71''E | Niedda Cave, medium to coarse sand, -26/-30 m | 04/09/2010 |
| 12b | Costa Paradiso | 41°3'8.84''N; 8°56'15.71''E | Niedda Cave, medium to coarse sand, -24/-34 m | 11/09/2010 |
| 12c | Costa Paradiso | 41°02'48.98''N; 8°56'9.25''E | Tinnari, retrodunal pond; salinity: 8‰. Algae at -0.5 m | 11/09/2010 |
| 13a | Capo Caccia | 40°33'37.83''N; 8°9'45.35''E | Nereo Cave (exterior), medium to coarse sand, - 35 m | 17/09/2010 |
| 13b | Capo Caccia | 40°33'37.73''N; 8°9'46.01''E | Nereo Cave (interior), very coarse sand with pebbles, - 32 m | 17/09/2010 |
