# Supplementary material for: Patterns of Diversity in Soft-Bodied Meiofauna: Dispersal Ability and Body Size Matter
Source: PLoS One. 2012 Mar 23;7(3):e33801. doi: 10.1371/journal.pone.0033801 (PMC3311549; doi:10.1371/journal.pone.0033801)
Supplement: Table S2 — Ecological and biological attributes (adult body length, distribution, endobenthic habitat) for each species included in the analyses. (DOC) [file pone.0033801.s003.doc]

Table S2. Ecological and biological attributes (adult body length, distribution, habitat) for each species included in the analyses.

| **Site** | **Group** | **Species** | **Size (mm)** | **Restricted to Mediterranean or North Sea - Baltic** | **New species** | **Dubious identification** | **Dispersing stage** | **Interstitial only** | **Partenogenetic only** |
| --- | --- | --- | --- | --- | --- | --- | --- | --- | --- |
| Sardinia | Acoela | Acoela sp (orange dot) | 3.000 | YES | YES | NO | NO | YES | NO |
| Sardinia | Acoela | Acoela sp (red pigment) | 0.600 | YES | YES | NO | NO | YES | NO |
| Sardinia | Acoela | Actinoposthia sp. | 2.000 | YES | YES | NO | NO | YES | NO |
| Sardinia | Acoela | Childia sp. 1 | 3.000 | YES | YES | NO | NO | YES | NO |
| Sardinia | Acoela | Childia sp. 2 | 3.000 | YES | YES | NO | NO | YES | NO |
| Sardinia | Acoela | Childia sp. 3 | 3.000 | YES | YES | NO | NO | YES | NO |
| Sardinia | Acoela | Childia sp. 4 | 3.000 | YES | YES | NO | NO | YES | NO |
| Sardinia | Acoela | Childia sp. 5 | 2.000 | YES | YES | NO | NO | YES | NO |
| Sardinia | Acoela | Diopisthoporus sp. | 0.700 | YES | YES | NO | NO | YES | NO |
| Sardinia | Acoela | Haplogonaria sp. 1 | 1.000 | YES | YES | NO | NO | YES | NO |
| Sardinia | Acoela | Haplogonaria sp. 2 | 0.800 | YES | YES | NO | NO | YES | NO |
| Sardinia | Acoela | Kuma sp. | 0.700 | YES | YES | NO | NO | YES | NO |
| Sardinia | Acoela | Otocelis sp. | 0.400 | YES | YES | NO | NO | YES | NO |
| Sardinia | Acoela | Paratomella rubra | 2.000 | NO | NO | NO | NO | YES | NO |
| Sardinia | Acoela | Philactinoposthia sp. 1 | 0.500 | YES | YES | NO | NO | YES | NO |
| Sardinia | Acoela | Philactinoposthia sp. 2 | 1.000 | YES | YES | NO | NO | YES | NO |
| Sardinia | Acoela | Philactinoposthia sp. 3 | 2.000 | YES | YES | NO | NO | YES | NO |
| Sardinia | Acoela | Philactinoposthia sp. 4 | 1.500 | YES | YES | NO | NO | YES | NO |
| Sardinia | Acoela | Proporus sp. | 1.500 | YES | YES | NO | NO | YES | NO |
| Sardinia | Acoela | Solenofilomorpha sp. 1 | 1.000 | YES | YES | NO | NO | YES | NO |
| Sardinia | Acoela | Solenofilomorpha sp. 2 | 2.000 | YES | YES | NO | NO | YES | NO |
| Sardinia | Acoela | Solenofilomorpha sp. 3 | 2.000 | NA | NA | YES | NO | YES | NO |
| Sardinia | Acoela | Symsagittifera corsicae | 4.000 | YES | NO | NO | NO | NO | NO |
| Sardinia | Annelida | Meganerilla sp. | 0.700 | NA | NA | YES | YES | NA | NO |
| Sardinia | Annelida | Mesonerilla intermedia | 1.200 | NO | NO | NO | NO | YES | NO |
| Sardinia | Annelida | Mesonerilla biantennata | 0.600 | NO | NO | NO | NO | YES | NO |
| Sardinia | Annelida | Mesonerilla armoricana | 1.000 | NO | NO | NO | NO | YES | NO |
| Sardinia | Annelida | Mesonerilla sp. | 1.000 | NA | NA | YES | NO | YES | NO |
| Sardinia | Annelida | Mesonerilla n. sp. | 0.800 | YES | YES | NO | NO | YES | NO |
| Sardinia | Annelida | Nerillidium mediterraneum | 0.600 | NO | NO | NO | NO | YES | NO |
| Sardinia | Annelida | Trochonerilla sp. | 0.500 | NA | NA | YES | NA | YES | NO |
| Sardinia | Annelida | Polygordius sp. | 5.000 | NA | NA | YES | YES | YES | NO |
| Sardinia | Annelida | Protodrilus gracilis | 6.500 | NO | NO | NO | YES | YES | NO |
| Sardinia | Annelida | Protodrilus purpureus | 13.000 | NO | NO | NO | YES | YES | NO |
| Sardinia | Annelida | Protodrilus similis | 2.000 | YES | NO | NO | YES | YES | NO |
| Sardinia | Annelida | Psammodrilus n. sp. | 2.500 | YES | YES | NO | NO | YES | NO |
| Sardinia | Gastrotricha | Acanthodasys aculeatus | 0.600 | NO | NO | NO | NO | YES | NO |
| Sardinia | Gastrotricha | Acanthodasys cf caribbeanensis | 0.606 | YES | NO | NO | NO | YES | NO |
| Sardinia | Gastrotricha | Aspidiophorus marinus | 0.172 | NO | NO | NO | NO | YES | YES |
| Sardinia | Gastrotricha | Aspidiophorus mediterraneus | 0.138 | NO | NO | NO | NO | YES | YES |
| Sardinia | Gastrotricha | Aspidiophorus paramediterraneus | 0.220 | NO | NO | NO | NO | YES | YES |
| Sardinia | Gastrotricha | Aspidiophorus tentaculatus | 0.330 | YES | NO | NO | NO | YES | YES |
| Sardinia | Gastrotricha | Cephalodasys n.sp.1It | 0.932 | YES | YES | NO | NO | YES | NO |
| Sardinia | Gastrotricha | Cephalodasys sp.2It | 0.848 | NA | NA | YES | NO | YES | NA |
| Sardinia | Gastrotricha | Chaetonotus aegilonensis | 0.169 | YES | NO | NO | NO | YES | YES |
| Sardinia | Gastrotricha | Chaetonotus apechochaetus | 0.140 | YES | NO | NO | NO | YES | YES |
| Sardinia | Gastrotricha | Chaetonotus atrox | 0.130 | NO | NO | NO | NO | YES | YES |
| Sardinia | Gastrotricha | Chaetonotus n.sp. 1It | 0.135 | YES | YES | NO | NO | YES | YES |
| Sardinia | Gastrotricha | Chaetonotus n.sp. 2It | 0.127 | YES | YES | NO | NO | YES | YES |
| Sardinia | Gastrotricha | Chaetonotus n.sp. 3It | 0.167 | YES | NA | YES | NO | YES | YES |
| Sardinia | Gastrotricha | Chaetonotus neptuni | 0.241 | NO | NO | NO | NO | YES | YES |
| Sardinia | Gastrotricha | Chaetonotus siciliensis | 0.201 | NO | NO | NO | NO | YES | YES |
| Sardinia | Gastrotricha | Crasiella n.sp.It | 0.500 | YES | YES | NO | NO | YES | NO |
| Sardinia | Gastrotricha | Dactylopodola typhle | 0.520 | NO | NO | NO | NO | YES | NO |
| Sardinia | Gastrotricha | Diplodasys sanctaemariae | 0.470 | YES | NO | NO | NO | YES | NO |
| Sardinia | Gastrotricha | Halichaetonotus euromarinus | 0.110 | NO | NO | NO | NO | YES | YES |
| Sardinia | Gastrotricha | Halichaetonotus n.sp.It | 0.101 | YES | YES | NO | NO | YES | YES |
| Sardinia | Gastrotricha | Heterolepidoderma n.sp.It | 0.096 | YES | YES | NO | NO | YES | NO |
| Sardinia | Gastrotricha | Heteroxenotrichula n.sp.It | 0.285 | YES | YES | NO | NO | YES | NO |
| Sardinia | Gastrotricha | Heteroxenotrichula pygmaea | 0.103 | NO | NO | NO | NO | YES | YES |
| Sardinia | Gastrotricha | Lepidodasys martini | 0.850 | NO | NO | NO | NO | YES | NO |
| Sardinia | Gastrotricha | Lepidodasys n.sp.It | 0.534 | YES | NA | YES | NO | YES | NO |
| Sardinia | Gastrotricha | Lepidodasys platyurus | 0.847 | YES | NO | NO | NO | YES | NO |
| Sardinia | Gastrotricha | Lepidodasys unicarenatus | 0.450 | YES | NO | NO | NO | YES | NO |
| Sardinia | Gastrotricha | Macrodasys n.sp.1It | 0.716 | YES | NA | YES | NO | YES | NO |
| Sardinia | Gastrotricha | Macrodasys sp. 2It | 0.526 | NA | NA | YES | NO | YES | NO |
| Sardinia | Gastrotricha | Megadasys n.sp.It | 1.978 | YES | YES | NO | NO | YES | NO |
| Sardinia | Gastrotricha | Mesodasys laticaudatus | 0.800 | NO | NO | NO | NO | YES | NO |
| Sardinia | Gastrotricha | Musellifer delamarei | 0.163 | YES | NO | NO | NO | YES | NO |
| Sardinia | Gastrotricha | n.gen.1 n.sp.1 It Dendro | 0.306 | YES | YES | NO | NO | YES | NO |
| Sardinia | Gastrotricha | n.gen.1 n.sp.1It Macro | 0.410 | YES | YES | NO | NO | YES | NO |
| Sardinia | Gastrotricha | Oregodasys ocellatus | 0.366 | NA | NO | NO | NO | YES | NO |
| Sardinia | Gastrotricha | Paradasys n.sp.It | 1.172 | YES | YES | NO | NO | YES | NO |
| Sardinia | Gastrotricha | Paratubanella n.sp.It | 1.010 | YES | NO | NO | NO | YES | NO |
| Sardinia | Gastrotricha | Paraturbanella dohrni | 0.559 | YES | NO | NO | NO | YES | NO |
| Sardinia | Gastrotricha | Paraturbanella pallida | 0.652 | NO | NO | NO | NO | YES | NO |
| Sardinia | Gastrotricha | Paraturbanella teissieri | 0.450 | NO | NO | NO | NO | YES | NO |
| Sardinia | Gastrotricha | Ptychostomella mediterranea | 0.180 | YES | NO | NO | NO | YES | NO |
| Sardinia | Gastrotricha | Ptychostomella n.sp. | 0.235 | YES | NO | NO | NO | YES | NO |
| Sardinia | Gastrotricha | Tetranchyoderma thysanophorum | 0.460 | YES | NO | NO | NO | YES | NO |
| Sardinia | Gastrotricha | Tetranchyroderma aapton | 0.265 | YES | NO | NO | NO | YES | NO |
| Sardinia | Gastrotricha | Tetranchyroderma cirrophorum | 0.647 | YES | NO | NO | NO | YES | NO |
| Sardinia | Gastrotricha | Tetranchyroderma heterotubulatum | 0.330 | NO | NO | NO | NO | YES | NO |
| Sardinia | Gastrotricha | Tetranchyroderma hirtum | 0.400 | YES | NO | NO | NO | YES | NO |
| Sardinia | Gastrotricha | Tetranchyroderma inaequitubulatum | 0.391 | YES | NO | NO | NO | YES | NO |
| Sardinia | Gastrotricha | Tetranchyroderma insulare | 0.280 | YES | NO | NO | NO | YES | NO |
| Sardinia | Gastrotricha | Tetranchyroderma n.sp.2It | 0.368 | YES | YES | NO | NO | YES | NO |
| Sardinia | Gastrotricha | Tetranchyroderma quadritentaculatum | 0.480 | YES | NO | NO | NO | YES | NO |
| Sardinia | Gastrotricha | Tetranchyroderma sp.1It | 0.202 | NA | NA | YES | NO | YES | NO |
| Sardinia | Gastrotricha | Tetranchyroderma symphorochetum | 0.369 | YES | NO | NO | NO | YES | NO |
| Sardinia | Gastrotricha | Tetranchyroderma thysanogaster | 0.487 | NO | NO | NO | NO | YES | NO |
| Sardinia | Gastrotricha | Thaumastoderma mediterraneum | 0.286 | YES | NO | NO | NO | YES | NO |
| Sardinia | Gastrotricha | Urodasys apuliensis | 0.702 | YES | NO | NO | NO | YES | NO |
| Sardinia | Gastrotricha | Urodasys sp3It | 0.475 | YES | YES | NO | NO | YES | NO |
| Sardinia | Gastrotricha | Urodasys viviparus | 0.368 | NO | NO | NO | NO | YES | YES |
| Sardinia | Gastrotricha | Xenotrichula puncata | 0.180 | NO | NO | NO | NO | YES | NO |
| Sardinia | Proseriata | Archilina deceptoria | 2.000 | YES | NO | NO | NO | YES | NO |
| Sardinia | Proseriata | Archilina n.sp. | 2.500 | YES | YES | NO | NO | YES | NO |
| Sardinia | Proseriata | Archimonocelis carmelitana | 8.000 | YES | NO | NO | NO | YES | NO |
| Sardinia | Proseriata | Archimonocelis meixneri | 5.000 | YES | NO | NO | NO | YES | NO |
| Sardinia | Proseriata | Archimonocelis n.sp. 1 | 6.000 | YES | YES | NO | NO | YES | NO |
| Sardinia | Proseriata | Archimonocelis n.sp. 2 | 6.000 | YES | YES | NO | NO | YES | NO |
| Sardinia | Proseriata | Archimonocelis staresoi | 5.000 | YES | NO | NO | NO | YES | NO |
| Sardinia | Proseriata | Boreocelis filicauda | 1.500 | NO | NO | NO | NO | YES | NO |
| Sardinia | Proseriata | Calviria sublittoralis | 5.000 | YES | NO | NO | NO | YES | NO |
| Sardinia | Proseriata | Coelogynopora gynocotyla | 7.000 | NO | NO | NO | NO | YES | NO |
| Sardinia | Proseriata | Coelogynopora n.sp. | 6.000 | YES | YES | NO | NO | YES | NO |
| Sardinia | Proseriata | Duplominona corsicana | 2.500 | YES | NO | NO | NO | YES | NO |
| Sardinia | Proseriata | Duplominona longicirrus | 2.850 | YES | NO | NO | NO | YES | NO |
| Sardinia | Proseriata | Duplominona n.sp. 1 | 2.000 | YES | YES | NO | NO | YES | NO |
| Sardinia | Proseriata | Duplominona n.sp. 2 | 2.000 | YES | YES | NO | NO | YES | NO |
| Sardinia | Proseriata | Duplominona n.sp. 3 | 2.000 | YES | YES | NO | NO | YES | NO |
| Sardinia | Proseriata | Monostichoplana n.sp. | 8.000 | YES | YES | NO | NO | YES | NO |
| Sardinia | Proseriata | Monotoplana diorchis | 1.500 | NO | NO | NO | YES | NO | NO |
| Sardinia | Proseriata | Nematoplana corsicana | 7.000 | YES | NO | NO | NO | YES | NO |
| Sardinia | Proseriata | Parotoplana geminispina | 2.500 | YES | NO | NO | NO | YES | NO |
| Sardinia | Proseriata | Parotoplana n.sp. 1 | 2.500 | YES | YES | NO | NO | YES | NO |
| Sardinia | Proseriata | Parotoplana n.sp. 2 | 2.500 | YES | YES | NO | NO | YES | NO |
| Sardinia | Proseriata | Parotoplana n.sp. 3 | 2.500 | YES | YES | NO | NO | YES | NO |
| Sardinia | Proseriata | Parotoplana n.sp. 4 | 2.500 | YES | YES | NO | NO | YES | NO |
| Sardinia | Proseriata | Parotoplana n.sp. 5 | 2.500 | YES | YES | NO | NO | YES | NO |
| Sardinia | Proseriata | Parotoplana pythagorae | 2.000 | YES | NO | NO | NO | YES | NO |
| Sardinia | Proseriata | Parotoplana renatae-sp. 1 | 2.500 | YES | YES | NO | NO | YES | NO |
| Sardinia | Proseriata | Parotoplana renatae-sp. 2 | 2.500 | YES | YES | NO | NO | YES | NO |
| Sardinia | Proseriata | Parotoplana spathifera | 2.500 | YES | NO | NO | NO | YES | NO |
| Sardinia | Proseriata | Parotoplanella n.sp. | 2.000 | YES | YES | NO | NO | YES | NO |
| Sardinia | Proseriata | Philosyrtis sp. | 1.000 | NA | NA | YES | NO | YES | NO |
| Sardinia | Proseriata | Polystyliphora n.sp. | 4.000 | YES | YES | NO | NO | YES | NO |
| Sardinia | Proseriata | Stilivannuccia n.sp. | 8.000 | YES | YES | NO | NO | YES | NO |
| Sardinia | Proseriata | Xenotoplana acus | 2.500 | YES | NO | NO | NO | YES | NO |
| Sardinia | Rhabdocoela | Ancistrorhynchus sp. (juv.) | 1.000 | NA | NA | YES | NA | NA | NO |
| Sardinia | Rhabdocoela | Austrorhynchus bruneti | 1.000 | YES | NO | NO | NO | YES | NO |
| Sardinia | Rhabdocoela | Austrorhynchus karlingi | 1.500 | YES | NO | NO | NO | YES | NO |
| Sardinia | Rhabdocoela | Balgetia?? sp. | 0.500 | NA | NA | YES | NA | NA | NO |
| Sardinia | Rhabdocoela | Baltoplana valkanovi | 1.800 | NO | NO | NO | NO | YES | NO |
| Sardinia | Rhabdocoela | Byrsophlebidae nov. sp. | 0.400 | YES | YES | NO | NO | NO | NO |
| Sardinia | Rhabdocoela | Carcharodorhynchus multidentatus | 1.100 | YES | NO | NO | NO | YES | NO |
| Sardinia | Rhabdocoela | Carcharodorhynchus nov. sp. | 2.100 | YES | YES | NO | NO | NO | NO |
| Sardinia | Rhabdocoela | Ceratopera gracilis | 0.800 | NO | NO | NO | NO | NO | NO |
| Sardinia | Rhabdocoela | Cheliplana asica / terminalis | 1.200 | NO | NO | NO | NO | YES | NO |
| Sardinia | Rhabdocoela | Cheliplana nov. sp. 1 | 1.200 | YES | YES | NO | NO | YES | NO |
| Sardinia | Rhabdocoela | Cheliplana nov. sp. 2 | 1.000 | YES | YES | NO | NO | YES | NO |
| Sardinia | Rhabdocoela | Cheliplana sp. 1 | 0.700 | NA | NA | YES | NA | NA | NO |
| Sardinia | Rhabdocoela | Cheliplana sp. 2 | 1.200 | NA | NA | YES | NA | NA | NO |
| Sardinia | Rhabdocoela | Ciliopharyngiella?? sp. | 2.700 | YES | NO | NO | NO | YES | NO |
| Sardinia | Rhabdocoela | Coronhelmis nov. sp. | 1.000 | YES | YES | NO | NO | YES | NO |
| Sardinia | Rhabdocoela | Cystiplana paradoxa | 1.700 | NO | NO | NO | NO | YES | NO |
| Sardinia | Rhabdocoela | Cystiplex axi | 1.300 | NO | NO | NO | NO | YES | NO |
| Sardinia | Rhabdocoela | Dalyellida sp. | 0.900 | NA | NA | YES | NA | NA | NO |
| Sardinia | Rhabdocoela | Djeziraia sp. | 1.200 | NA | NA | YES | NA | NA | NO |
| Sardinia | Rhabdocoela | Duplacrorhynchus megalophallus | 0.800 | YES | NO | NO | NO | YES | NO |
| Sardinia | Rhabdocoela | Gallorhynchus nov. sp. | 0.600 | YES | YES | NO | NO | YES | NO |
| Sardinia | Rhabdocoela | Gyratrix hermaphroditus | 1.000 | NO | NO | NO | NO | NO | NO |
| Sardinia | Rhabdocoela | Haplovejdovskya nov. sp. | 0.700 | YES | YES | NO | NO | YES | NO |
| Sardinia | Rhabdocoela | Itaipusa nov. sp. | 2.800 | YES | YES | NO | NO | YES | NO |
| Sardinia | Rhabdocoela | Kalyptorhynch sp. | NA | NA | NA | YES | NA | NA | NO |
| Sardinia | Rhabdocoela | Kytorhynchidae sp. | 0.500 | NA | NA | YES | NA | NA | NO |
| Sardinia | Rhabdocoela | Kytorhynchus nov. sp. | 0.900 | YES | YES | NO | NO | YES | NO |
| Sardinia | Rhabdocoela | Lagenopolycystis nov. sp. | 0.900 | YES | YES | NO | NO | YES | NO |
| Sardinia | Rhabdocoela | Limipolycystis nov. sp. 1 | 0.500 | YES | YES | NO | NO | YES | NO |
| Sardinia | Rhabdocoela | Limipolycystis nov. sp. 2 | 1.000 | YES | YES | NO | NO | YES | NO |
| Sardinia | Rhabdocoela | Limipolycystis nov. sp. 3 | 0.700 | YES | YES | NO | NO | YES | NO |
| Sardinia | Rhabdocoela | Paulodora contorta | 1.000 | NO | NO | NO | NO | YES | NO |
| Sardinia | Rhabdocoela | Paulodora nov. sp. | 0.700 | YES | YES | NO | NO | NO | NO |
| Sardinia | Rhabdocoela | Polycystis naegelii | 1.100 | NO | NO | NO | NO | NO | NO |
| Sardinia | Rhabdocoela | Progyrator mamertinus | 1.400 | NO | NO | NO | NO | NO | NO |
| Sardinia | Rhabdocoela | Promesostoma ensifer | 0.600 | NO | NO | NO | NO | NO | NO |
| Sardinia | Rhabdocoela | Promesostoma maculosum | 1.700 | NO | NO | NO | NO | YES | NO |
| Sardinia | Rhabdocoela | Promesostoma nov. sp. | 0.500 | YES | YES | NO | NO | YES | NO |
| Sardinia | Rhabdocoela | Promesostomidae nov. sp. 1 | 1.300 | YES | YES | NO | NO | YES | NO |
| Sardinia | Rhabdocoela | Promesostomidae nov. sp. 2 | NA | YES | YES | NO | NO | YES | NO |
| Sardinia | Rhabdocoela | Psammopolycystis nov. sp. | 0.600 | YES | YES | NO | NO | YES | NO |
| Sardinia | Rhabdocoela | Rogneda colpaerti | 0.700 | YES | NO | NO | NO | YES | NO |
| Sardinia | Rhabdocoela | Solenopharyngide sp. 1 | NA | NA | NA | YES | NA | NA | NO |
| Sardinia | Rhabdocoela | Solenopharyngide sp. 2 | 0.300 | NA | NA | YES | NA | NA | NO |
| Sardinia | Rhabdocoela | Solenopharyngide sp. 3 | 0.400 | NA | NA | YES | NA | NA | NO |
| Sardinia | Rhabdocoela | Trigonostomum australis | 1.300 | NO | NO | NO | NO | NO | NO |
| Sardinia | Rhabdocoela | Trigonostomum penicillatum | 0.700 | NO | NO | NO | NO | NO | NO |
| Sardinia | Rhabdocoela | Trigonostomum setigerum | 0.800 | NO | NO | NO | NO | NO | NO |
| Sardinia | Rhabdocoela | Trigonostomum venenosum | 0.700 | NO | NO | NO | NO | NO | NO |
| Sardinia | Rhabdocoela | Typhloplanidae sp. 1 | 1.100 | NA | NA | YES | NA | NA | NO |
| Sardinia | Rhabdocoela | Typhloplanidae sp. 2 | 1.300 | NA | NA | YES | NA | NA | NO |
| Sardinia | Rhabdocoela | Polycystididae nov. gen. nov. sp. | 0.700 | NO | YES | NO | NO | YES | NO |
| Sardinia | Rhabdocoela | Typhlopolycystis nov. sp. 1 | 0.600 | YES | YES | NO | NO | YES | NO |
| Sardinia | Rhabdocoela | Typhlopolycystis nov. sp. 2 | 0.500 | YES | YES | NO | NO | YES | NO |
| Sardinia | Rotifera | Brachionus ibericus | 0.220 | NO | NO | NO | YES | NO | NO |
| Sardinia | Rotifera | Brachionus urceolaris | 0.300 | NO | NO | NO | YES | NO | NO |
| Sardinia | Rotifera | Colurella colurus | 0.110 | NO | NO | NO | YES | NO | NO |
| Sardinia | Rotifera | Colurella dicentra | 0.120 | NO | NO | NO | YES | NO | NO |
| Sardinia | Rotifera | Colurella sp. | 0.120 | NA | NA | YES | YES | NO | NO |
| Sardinia | Rotifera | Encentrum sp. | 0.350 | NA | NA | YES | YES | NO | NO |
| Sardinia | Rotifera | Eosphora ehrenbergi | 0.450 | NO | NO | NO | YES | NO | NO |
| Sardinia | Rotifera | Lecane bulla | 0.210 | NO | NO | NO | YES | NO | NO |
| Sardinia | Rotifera | Lepadella sp. | 0.080 | NA | NA | YES | YES | NO | NO |
| Sardinia | Rotifera | Proales halophila | 0.180 | NO | NO | NO | YES | NO | NO |
| Sardinia | Rotifera | Proales similis | 0.180 | NO | NO | NO | YES | NO | NO |
| Sardinia | Rotifera | Proales sp. | 0.180 | NA | NA | YES | YES | NO | NO |
| Sardinia | Rotifera | Rotaria laticeps | 0.400 | YES | NO | NO | YES | NO | YES |
| Sardinia | Rotifera | Rotaria sp. | 0.450 | NA | NA | YES | YES | NO | YES |
| Sardinia | Rotifera | Testudinella clypeata | 0.170 | NO | NO | NO | YES | NO | NO |
| Sardinia | Rotifera | Testudinella obscura | 0.136 | NO | NO | NO | YES | NO | NO |
| Sweden | Acoela | Acoela sp (with scleritic inclusions) | 1.000 | YES | YES | NO | NO | YES | NO |
| Sweden | Acoela | Anaperus rubellus | 1.500 | NO | NO | NO | NO | YES | NO |
| Sweden | Acoela | Archaphanstoma macrospiriferum | 0.800 | YES | NO | NO | NO | YES | NO |
| Sweden | Acoela | Archocelis macrorhabditis | 0.800 | YES | NO | NO | NO | YES | NO |
| Sweden | Acoela | Childia macroposthium | 1.500 | NO | NO | NO | NO | YES | NO |
| Sweden | Acoela | Childia submaculatum | 1.500 | NO | NO | NO | NO | YES | NO |
| Sweden | Acoela | Childia triangulifera | 3.000 | NO | NO | NO | NO | YES | NO |
| Sweden | Acoela | Diopisthoporus longitubus | 1.200 | NO | NO | NO | NO | YES | NO |
| Sweden | Acoela | Endocincta punctata | 0.900 | NO | NO | NO | NO | YES | NO |
| Sweden | Acoela | Haplogonaria viridis | 0.800 | NO | NO | NO | NO | YES | NO |
| Sweden | Acoela | Haploposthia lactomaculata | 3.000 | YES | NO | NO | NO | YES | NO |
| Sweden | Acoela | Haploposthia rubropunctata | 1.000 | NO | NO | NO | NO | YES | NO |
| Sweden | Acoela | Mecynostomidae sp. | 1.000 | YES | YES | NO | NO | YES | NO |
| Sweden | Acoela | Mecynostomum sp. | 0.800 | YES | YES | NO | NO | YES | NO |
| Sweden | Acoela | Notocelis gullmarensis | 0.500 | NO | NO | NO | NO | YES | NO |
| Sweden | Acoela | Paedomecynostomum sp. | 0.800 | YES | YES | NO | NO | YES | NO |
| Sweden | Acoela | Paramecynostomum diversicolor | 0.600 | NO | NO | NO | NO | YES | NO |
| Sweden | Acoela | Philactinoposthia saliens | 1.000 | NO | NO | NO | NO | YES | NO |
| Sweden | Acoela | Philactinoposthia sp. 1 | 1.000 | YES | YES | NO | NO | YES | NO |
| Sweden | Acoela | Pseudmecynostmum bruneum | 1.800 | YES | NO | NO | NO | YES | NO |
| Sweden | Acoela | Pseudmecynostomum sp. | 1.000 | YES | YES | NO | NO | YES | NO |
| Sweden | Annelida | Protodrilus rubropharyngeus | 9.000 | NO | NO | NO | YES | YES | NO |
| Sweden | Annelida | Protodrilus helgolandicus | 9.300 | NO | NO | NO | YES | YES | NO |
| Sweden | Annelida | Protodrilus adhaerens | 3.500 | NO | NO | NO | YES | YES | NO |
| Sweden | Annelida | Protodrilus oculifer | 4.300 | NO | NO | NO | YES | YES | NO |
| Sweden | Annelida | Trilobodrilus axi | 0.900 | NO | NO | NO | NO | YES | NO |
| Sweden | Annelida | Diurodrilus minimus | 0.300 | NO | NO | NO | NO | YES | NO |
| Sweden | Gastrotricha | Acanthodasys aculeatus | 0.600 | NO | NO | NO | NO | YES | NO |
| Sweden | Gastrotricha | Aspidiophorus marinus | 0.172 | NO | NO | NO | NO | YES | NO |
| Sweden* | Gastrotricha | Aspidiophorus n.sp. | 0.118 | YES | YES | NO | NO | YES | YES |
| Sweden* | Gastrotricha | Cephalodasys maximus | 0.652 | NO | NO | NO | NO | YES | NO |
| Sweden* | Gastrotricha | Cephalodasys turbanelloides | 0.928 | NO | NO | NO | NO | YES | NO |
| Sweden | Gastrotricha | Chaetonotus atrox | 0.130 | NO | NO | NO | NO | YES | YES |
| Sweden* | Gastrotricha | Chaetonotus dispar | 0.126 | NA | NO | NO | NO | YES | YES |
| Sweden | Gastrotricha | Chaetonotus inaequidentatus | 0.137 | NO | NO | NO | NO | YES | YES |
| Sweden | Gastrotricha | Chaetonotus sp.1S | 0.148 | YES | YES | NO | NO | YES | YES |
| Sweden | Gastrotricha | Chaetonotus tempestivus | 0.127 | YES | NO | NO | NO | YES | YES |
| Sweden | Gastrotricha | Chaetonotus variosquamatus | 0.147 | NO | NO | NO | NO | YES | YES |
| Sweden | Gastrotricha | Crasiella diplura | 0.554 | YES | NO | NO | NO | YES | NO |
| Sweden | Gastrotricha | Dinodasys mirabilis | 0.320 | YES | NO | NO | NO | YES | NO |
| Sweden | Gastrotricha | Diplodasys ankeli | 0.481 | NO | NO | NO | NO | YES | NO |
| Sweden | Gastrotricha | Dolichodasys elongatus | 1.903 | NO | NO | NO | NO | YES | NO |
| Sweden | Gastrotricha | Hachaetonotus aculifer | 0.151 | NO | NO | NO | NO | YES | YES |
| Sweden | Gastrotricha | Hachaetonotus atlanticus | 0.183 | NO | NO | NO | NO | YES | YES |
| Sweden | Gastrotricha | Halichaetonotus paradoxus | 0.140 | NO | NO | NO | NO | YES | YES |
| Sweden | Gastrotricha | Halichaetonotus somniculosus | 0.134 | YES | NO | NO | NO | YES | YES |
| Sweden | Gastrotricha | Halichaetontous sp.1S | 0.147 | YES | YES | NO | NO | YES | YES |
| Sweden | Gastrotricha | Halichaetontous sp.2S | 0.135 | YES | YES | NO | NO | YES | YES |
| Sweden* | Gastrotricha | Halichetonotus euromarinus | 0.120 | NO | NO | NO | NO | YES | YES |
| Sweden* | Gastrotricha | Halichetonotus margaretae | 0.144 | NO | NO | NO | NO | YES | YES |
| Sweden | Gastrotricha | Lepidodasys martini | 0.850 | NO | NO | NO | NO | YES | NO |
| Sweden | Gastrotricha | Lepidodasys platyurus | 0.847 | NO | NO | NO | NO | YES | NO |
| Sweden | Gastrotricha | Lepidodasys sp. 1S | 0.745 | YES | YES | NO | NO | YES | NO |
| Sweden | Gastrotricha | Macrodasys sp.1S | 0.921 | YES | YES | NO | NO | YES | NO |
| Sweden | Gastrotricha | Macrodasys sp.2S | 0.875 | YES | YES | NO | NO | YES | NO |
| Sweden* | Gastrotricha | Macrodasys sp.3S | 0.620 | NA | NA | NA | NO | YES | NO |
| Sweden* | Gastrotricha | Megadasys sp1-krist | 1.700 | YES | YES | NO | NO | YES | NO |
| Sweden | Gastrotricha | Mesodasys laticaudatus | 1.005 | NO | NO | NO | NO | YES | NO |
| Sweden* | Gastrotricha | n.g.1 n.sp.2S | 0.589 | YES | YES | NO | NO | YES | NO |
| Sweden | Gastrotricha | Paradasys subterraneus | 0.467 | NO | NO | NO | NO | YES | NO |
| Sweden* | Gastrotricha | Paraturbanella sp1 | 0.616 | YES | YES | NO | NO | YES | NO |
| Sweden | Gastrotricha | Tetranchyroderma megastomum | 0.400 | YES | NO | NO | NO | YES | NO |
| Sweden | Gastrotricha | Tetranchyroderma sp. 2S | 0.218 | YES | YES | NO | NO | YES | NO |
| Sweden | Gastrotricha | Tetranchyroderma suecica | 0.450 | YES | NO | NO | NO | YES | NO |
| Sweden | Gastrotricha | Thaumastoderma heideri | 0.240 | NO | NO | NO | NO | YES | NO |
| Sweden | Gastrotricha | Thaumastoderma moebjergi | 0.204 | YES | NO | NO | NO | YES | NO |
| Sweden | Gastrotricha | Turbanella cornuta | 0.735 | NO | NO | NO | NO | YES | NO |
| Sweden | Gastrotricha | Turbanella lutheri | 0.801 | YES | NO | NO | NO | YES | NO |
| Sweden | Gastrotricha | Urodasys mirabilis | 0.615 | YES | NO | NO | NO | YES | NO |
| Sweden | Gastrotricha | Xenotrichula punctata | 0.180 | NO | NO | NO | NO | YES | NO |
| Sweden | Proseriata | Archiloa westbladi | 4.000 | YES | NO | NO | NO | YES | NO |
| Sweden | Proseriata | Archilopsis spinosa | 3.500 | YES | NO | NO | NO | YES | NO |
| Sweden | Proseriata | Archilopsis unipunctata | 3.500 | YES | NO | NO | NO | YES | NO |
| Sweden | Proseriata | Boreocelis juv. cf filicauda | 1.500 | NO | NO | NO | NO | YES | NO |
| Sweden | Proseriata | Coelogynopora biarmata | 8.000 | NO | NO | NO | NO | YES | NO |
| Sweden | Proseriata | Coelogynopora gynocotyla | 7.000 | NO | NO | NO | NO | YES | NO |
| Sweden | Proseriata | Coelogynopora n. sp. | 5.000 | YES | YES | NO | NO | YES | NO |
| Sweden | Proseriata | Duplominona septentrionalis | 1.800 | YES | NO | NO | NO | YES | NO |
| Sweden | Proseriata | Itaspiella helgolandica | 1.600 | YES | NO | NO | NO | YES | NO |
| Sweden | Proseriata | Monocelididae n. gen. n. sp. | 1.300 | YES | YES | NO | NA | NO | NO |
| Sweden | Proseriata | Monocelis fusca | 3.000 | YES | NO | NO | NO | NO | NO |
| Sweden | Proseriata | Monocelis lineata “pigmented” | 3.000 | NO | NA | NO | NO | NO | NO |
| Sweden | Proseriata | Monocelis lineata “unpigmented” | 3.000 | NO | NA | NO | NO | YES | NO |
| Sweden | Proseriata | Monocelis n. sp. | 3.000 | YES | YES | NO | NO | NO | NO |
| Sweden | Proseriata | Monocelopsis otoplanoides | 2.000 | YES | NO | NO | NO | YES | NO |
| Sweden | Proseriata | Monostichoplana juv cf filum | 8.000 | YES | NO | NO | NO | YES | NO |
| Sweden | Proseriata | Monotoplana diorchis | 1.500 | NO | NO | NO | YES | NO | NO |
| Sweden | Proseriata | Philosyrtis fennica | 0.900 | YES | NO | NO | NO | YES | NO |
| Sweden | Proseriata | Promonotus schultzei | 3.500 | NO | NO | NO | NO | YES | NO |
| Sweden | Proseriata | Pseudorthoplana foliacea | 2.500 | NO | NO | NO | NO | YES | NO |
| Sweden | Proseriata | Pseudosyrtis subterranea | 1.000 | NO | NO | NO | NO | YES | NO |
| Sweden | Rhabdocoela | Acrorhynchides caledonicus | 2.000 | NO | NO | NO | NO | NO | NO |
| Sweden | Rhabdocoela | Byrsophlebs dubia | 0.500 | NO | NO | NO | NO | YES | NO |
| Sweden | Rhabdocoela | Carcharodorhynchus subterraneus | 1.200 | NO | NO | NO | NO | YES | NO |
| Sweden | Rhabdocoela | Cheliplana rubescens | 1.100 | NO | NO | NO | NO | YES | NO |
| Sweden | Rhabdocoela | Cheliplanilla caudata | 1.000 | NO | NO | NO | NO | YES | NO |
| Sweden | Rhabdocoela | Cicerina bervicirrus | 1.300 | YES | NO | NO | NO | YES | NO |
| Sweden | Rhabdocoela | Cytocystis clitellatus | 1.500 | YES | NO | NO | NO | YES | NO |
| Sweden | Rhabdocoela | Doliopharynx geminocirro | 1.000 | YES | NO | NO | NO | YES | NO |
| Sweden | Rhabdocoela | Ethmorhynchus anopthalmus | NA | NO | NO | NO | NO | YES | NO |
| Sweden | Rhabdocoela | Gyratrix hermaphroditus | 1.000 | NO | NO | NO | NO | NO | NO |
| Sweden | Rhabdocoela | Lenopharynx bathos | NA | YES | NO | NO | NO | YES | NO |
| Sweden | Rhabdocoela | Mariplanella frisia | 1.100 | YES | NO | NO | NO | YES | NO |
| Sweden | Rhabdocoela | Mesorhynchus terminostylus | 2.000 | NO | NO | NO | NO | YES | NO |
| Sweden | Rhabdocoela | Odontorhynchus aculeatus | 1.000 | YES | NO | NO | NO | YES | NO |
| Sweden | Rhabdocoela | Paragnathorhynchus subterraneus | 2.000 | YES | NO | NO | NO | YES | NO |
| Sweden | Rhabdocoela | Paulodora contorta | 1.000 | NO | NO | NO | NO | YES | NO |
| Sweden | Rhabdocoela | Phonorhynchus helgolandicus | 1.700 | NO | NO | NO | NO | NO | NO |
| Sweden | Rhabdocoela | Polycystididae nov. sp. | NA | YES | YES | NO | NO | YES | NO |
| Sweden | Rhabdocoela | Promesostoma marmoratum | 1.100 | NO | NO | NO | NO | NO | NO |
| Sweden | Rhabdocoela | Promesostoma neglectum | 1.500 | YES | NO | NO | NO | NO | NO |
| Sweden | Rhabdocoela | Promesostoma rostratum | 1.000 | NO | NO | NO | NO | NO | NO |
| Sweden | Rhabdocoela | Promesostomidae nov. sp. | NA | YES | YES | NO | NO | YES | NO |
| Sweden | Rhabdocoela | Proschizorhynchus gullmarensis | 3.000 | NO | NO | NO | NO | YES | NO |
| Sweden | Rhabdocoela | Provortex karlingi | 1.000 | NO | NO | NO | NO | NO | NO |
| Sweden | Rhabdocoela | Provortex tubiferus | 0.800 | YES | NO | NO | NO | NO | NO |
| Sweden | Rhabdocoela | Proxenetes quinquespinosus | 0.800 | YES | NO | NO | NO | YES | NO |
| Sweden | Rhabdocoela | Proxenetes trigonus | 0.600 | YES | NO | NO | NO | YES | NO |
| Sweden | Rhabdocoela | Psammorhynchus tubulipenis | 1.000 | YES | NO | NO | NO | YES | NO |
| Sweden | Rhabdocoela | Rogneda anglica | 1.500 | NO | NO | NO | NO | YES | NO |
| Sweden | Rhabdocoela | Scanorhynchus forcipatus | 0.800 | NO | NO | NO | NO | YES | NO |
| Sweden | Rhabdocoela | Solenopharyngidae sp. | NA | NA | NA | YES | NA | NA | NO |
| Sweden | Rhabdocoela | Trigonostomum armatum | 1.500 | NO | NO | NO | NO | NO | NO |
| Sweden | Rhabdocoela | Trigonostomum venenosum | 0.700 | NO | NO | NO | NO | NO | NO |
| Sweden | Rhabdocoela | Typhlopolycystididae nov. gen. nov. sp. | 0.700 | NO | YES | NO | NO | YES | NO |
| Sweden | Rhabdocoela | Zonorhynchus seminascatus | 2.500 | YES | NO | NO | NO | YES | NO |
| Sweden | Rotifera | Aspelta clydona | 0.290 | NO | NO | NO | YES | NO | NO |
| Sweden | Rotifera | Cephalodella sp. | 0.150 | NA | NA | YES | YES | NO | NO |
| Sweden | Rotifera | Colurella adriatica | 0.113 | NO | NO | NO | YES | NO | NO |
| Sweden | Rotifera | Colurella colurus | 0.110 | NO | NO | NO | YES | NO | NO |
| Sweden | Rotifera | Colurella dicentra | 0.120 | NO | NO | NO | YES | NO | NO |
| Sweden | Rotifera | Encentrum algente | 0.360 | NO | NO | NO | YES | NO | NO |
| Sweden | Rotifera | Encentrum bidentatum | 0.220 | YES | NO | NO | YES | NO | NO |
| Sweden | Rotifera | Encentrum limicola | 0.250 | NO | NO | NO | YES | NO | NO |
| Sweden | Rotifera | Encentrum marinum | 0.200 | NO | NO | NO | YES | NO | NO |
| Sweden | Rotifera | Encentrum sp. | 0.200 | NA | NA | YES | YES | NO | NO |
| Sweden | Rotifera | Lindia gravitata | 1.260 | YES | NO | NO | YES | NO | NO |
| Sweden | Rotifera | Lindia tecusa | 1.500 | NO | NO | NO | YES | NO | NO |
| Sweden | Rotifera | Notholca bipalium/liepetterseni | 0.240 | NO | NO | NO | YES | NO | NO |
| Sweden | Rotifera | Proales halophila | 0.180 | NO | NO | NO | YES | NO | NO |
| Sweden | Rotifera | Proales reinhardti | 0.380 | NO | NO | NO | YES | NO | NO |
| Sweden | Rotifera | Proales syltensis | 0.150 | YES | NO | NO | YES | NO | NO |
| Sweden | Rotifera | Synchaeta baltica | 0.523 | NO | NO | NO | YES | NO | NO |
| Sweden | Rotifera | Synchaeta cecilia | 0.188 | NO | NO | NO | YES | NO | NO |
| Sweden | Rotifera | Synchaeta gyrina | 0.326 | NO | NO | NO | YES | NO | NO |
| Sweden | Rotifera | Synchaeta vorax | 0.400 | NO | NO | NO | YES | NO | NO |
| Sweden | Rotifera | Testudinella clypeata | 0.170 | NO | NO | NO | YES | NO | NO |
| Sweden | Rotifera | Testudinella elliptica | 0.210 | NO | NO | NO | YES | NO | NO |
| Sweden | Rotifera | Testudinella obscura | 0.136 | NO | NO | NO | YES | NO | NO |

* from sites not reported in Willems et al [4].
