# Supplementary material for: Patterns of Diversity in Soft-Bodied Meiofauna: Dispersal Ability and Body Size Matter
Source: PLoS One. 2012 Mar 23;7(3):e33801. doi: 10.1371/journal.pone.0033801 (PMC3311549; doi:10.1371/journal.pone.0033801)
Supplement: Tables S3 — Acoela and Nemertodermatida. Species list and occurrence in Northern Sardinia. (DOC) [file pone.0033801.s004.doc]

**Table S3.**  Acoela and Nemertodermatida. Species list and occurrence in Northern Sardinia.

| **Taxon** | **Station** |
| --- | --- |
| **ACOELA** |  |
| **Actinoposthiidae** |  |
| *Actinoposthia* n.sp. 1 | 3 |
| *Philactinoposthia* n.sp. 1 | 1 |
| *Philactinoposthia* n.sp. 2 | 1;3 |
| *Philactinoposthia* n.sp. 3 | 1 |
| *Philactinoposthia* n.sp. 4 | 3 |
| **Childiidae** |  |
| *Childia* n.sp. 1 | 3 |
| *Childia* n.sp. 2 | 3 |
| *Childia* n.sp. 3 | 3 |
| *Childia* n.sp. 4 | 10 |
| *Childia* n.sp. 5 | 12a,b |
| **Diopisthoporidae** |  |
| *Diopisthoporus* n.sp. 1 | 12a,b |
| **Haploposthiidae** |  |
| *Haplogonaria* n.sp. 1 | 3 |
| *Haplogonaria* n.sp. 2 | 1 |
| *Kuma* n.sp. | 1 |
| **Otocelididae** |  |
| *Otocelis* n.sp. | 1 |
| **Paratomellidae** |  |
| *Paratomella rubra* Rieger & Ott, 1971 | 12b |
| **Proporidae** |  |
| *Proporus* n.sp. | 12a,b |
| **Sagittiferidae** |  |
| *Symsagittifera corsicae* Gschwentner, Baric & Rieger, 2002 | 3 |
| **Solenofilomorphidae** |  |
| *Solenofilomorfa* n.sp. 1 | 1;3;10;12a,b |
| *Solenofilomorfa* n.sp. 2 | 11b |
| *Solenofilomorfa* n.sp. 3 | 11b |
| **Incertae Saedis** |  |
| Acoela sp. 1 (orange dot) | 12b |
| Acoela sp. 2 (red pigment) | 1 |
| **Nemertodermatida** |  |
| **Ascopariidae** |  |
| *Flagellophora* sp. | 1;3;12a,b |
| **Nemertodermatidae** |  |
| *Nemertinoides elongatus* Riser, 1987 | 3 |
| *Nemertoderma* sp. | 1 |
| *Sterreria* sp. (unpigmented) | 1;12a,b |
| *Sterreria* sp. (red) | 1;3;12a,b |

Refer to Table S1 for the identification of sampling stations.
