# Supplementary material for: Patterns of Diversity in Soft-Bodied Meiofauna: Dispersal Ability and Body Size Matter
Source: PLoS One. 2012 Mar 23;7(3):e33801. doi: 10.1371/journal.pone.0033801 (PMC3311549; doi:10.1371/journal.pone.0033801)
Supplement: Tables S4 — Proseriata. Species list and occurrence in Northern Sardinia. (DOC) [file pone.0033801.s005.doc]

**Table S4.**  Proseriata. Species list and occurrence in Northern Sardinia.

| **Taxon** | **Station** |
| --- | --- |
| **Unguiphora** |  |
| *Polystyliphora* n.sp. | 12b |
| *Nematoplana corsicana* Curini-Galletti & Martens, 1992 | 1 |
| **Lithophora** |  |
| **Coelogynoporidae** |  |
| *Coelogynopora* cf *gynocotyla* Steinböck, 1924 | 1 |
| *Coelogynopora* n.sp. | 12b |
| *Stilivannuccia* n.sp. | 3 |
| **Calviriidae** |  |
| *Calviria sublittoralis* Martens & Curini-Galletti, 1993 | 3 |
| **Archimonocelididae** | 1;12b |
| *Archimonocelis staresoi* Martens & Curini-Galletti, 1993 | 2 |
| *Archimonocelis meixneri* Martens & Curini-Galletti, 1993 | 1;12a,b |
| *Archimonocelis carmelitana* Martens & Curini-Galletti, 1993 | 1;12a,b |
| *Archimonocelis* n.sp. 1 | 1 |
| *Archimonocelis* n.sp. 2 | 1;3;12b |
| **Otoplanidae** |  |
| *Xenotoplana acus* Ax, Weidemann & Ehlers, 1978 | 2;12a,b |
| *Monostichoplana* n.sp. | 3 |
| *Parotoplanella* n.sp. | 5a |
| *Parotoplana pythagorae* Delogu & Curini-Galletti, 2007 | 3 |
| *Parotoplana geminispina* Delogu & Curini-Galletti 2009 | 3 |
| *Parotoplana spathifera* Delogu & Curini-Galletti, 2007 | 12b |
| *Parotoplana* n.sp. 1 | 3 |
| *Parotoplana* n.sp. 2 | 1 |
| *Parotoplana* n.sp. 3 | 12a,b |
| *Parotoplana* n.sp. 4 | 12a,b |
| *Parotoplana* n.sp. 5 | 12a,b |
| *Parotoplana renatae/macrostyla* complex sp. 1 | 3;5a |
| *Parotoplana renatae/macrostyla* complex sp. 2 | 10;12b |
| *Philosyrtis* sp. | 1 |
| **Monocelididae** |  |
| *Duplominona corsicana* Martens, 1984 | 10;12a,b |
| *Duplominona longicirrus* Martens, 1984 | 12a,b |
| *Duplominona* n.sp. 1 | 12b |
| *Duplominona* n.sp. 2 | 1 |
| *Duplominona* n.sp. 3 | 1 |
| *Archilina deceptoria* Martens & Curini-Galletti, 1994 | 1;3;12a,b |
| *Archilina* n.sp. | 3 |
| *Boreocelis* cf *filicauda* Westblad, 1952 | 12a,b |
| *Monotoplana* cf *diorchis* Meixner, 1938 | 1;12a,b |

Refer to Table S1 for the identification of sampling stations.
