# Supplementary material for: Patterns of Diversity in Soft-Bodied Meiofauna: Dispersal Ability and Body Size Matter
Source: PLoS One. 2012 Mar 23;7(3):e33801. doi: 10.1371/journal.pone.0033801 (PMC3311549; doi:10.1371/journal.pone.0033801)
Supplement: Tables S5 — Rhabdocoela. Species list and occurrence in Northern Sardinia. (DOC) [file pone.0033801.s006.doc]

**Table S5.**  Rhabdocoela. Species list and occurrence in Northern Sardinia.

| **Taxon** | **Station** |
| --- | --- |
| **Kalyptorhynchia** |  |
| **Polycystididae** |  |
| *Austrorhynchus bruneti* Karling, 1977 | 1 |
| *Austrorhynchus karlingi* Brunet, 1965 | 1 |
| *Djeziraia* sp. | 4b |
| *Duplacrorhynchus megalophallus* Artois & Schockaert, 1999 | 4b;12b |
| *Gallorhynchus* n.sp. | 12b |
| *Gyratrix hermaphroditus* Ehrenberg, 1831 | 1;2;4b;5b;10;11b;12b,c |
| *Lagenopolycystis* n. sp. | 1;12b |
| *Limipolycystis* n.sp. 1 | 1 |
| *Limipolycystis* n.sp. 2 | 1 |
| *Limipolycystis* n.sp. 3 | 1 |
| *Paulodora contorta* (Schockaert & Karling, 1975) | 3 |
| *Paulodora* n.sp. | 11a |
| *Polycystis naegelii* Kölliker, 1845 | 5b;11a |
| *Progyrator mamertinus* (Graff, 1874) | 3 |
| *Psammopolycystis* n.sp. | 1 |
| *Rogneda colpaerti* Artois, 2008 | 1 |
| Polycystididae n.gen. n.sp. | 1;12b |
| *Typhlopolycystis* n.sp. 1 | 12a,b |
| *Typhlopolycystis* n.sp. 2 | 12a |
| **Gnathorhynchidae** |  |
| *Ancistrorhynchus* sp. (juv.) | 2;12b |
| **Karkinorhynchidae** |  |
| *Baltoplana valkanovi* Ax, 1959 | 3 |
| *Cheliplana asica terminalis* Brunet, 1968 | 10 |
| *Cheliplana* n.sp. 1 | 1 |
| *Cheliplana* n.sp. 2 | 10 |
| *Cheliplana* sp. 1 | 10 |
| *Cheliplana* sp. 2 | 10;12b |
| **Schizorhynchidae** |  |
| *Carcharodorhynchus* n.sp. | 3;5b |
| *Carcharodorhynchus multidentatus* Brunet, 1979 | 12b |
| **Cystiplanidae** |  |
| *Cystiplana paradoxa* Karling, 1964 | 3;12b |
| *Cystiplex axi* Karling, 1964 | 1;3;4b;10;11b |
| **Koinocystididae** |  |
| *Itaipusa* n.sp. | 11b |
| Koinocystididae sp. | 2 |
| **Dalytyphloplanida** |  |
| **Provorticidae** |  |
| *Balgetia*?? sp. | 2 |
| *Haplovejdovskya* n.sp. | 12b |
| Dalyellida sp. | 1 |
| **Byrsophlebidae** |  |
| *Byrsophlebidae* n.sp. | 5b |
| **Kytorhynchidae** |  |
| Kytorhynchidae sp. | 12a |
| *Kytorhynchus* n.sp. | 12b |
| **Promesostomidae** |  |
| *Coronhelmis* n.sp. | 2;10 |
| *Promesostoma ensifer* (Uljanin, 1870) | 3 |
| *Promesostoma maculosum* Ax, 1956 | 4b |
| *Promesostoma* n.sp. | 12b |
| Promesostomidae n.sp. 1 | 1 |
| Promesostomidae n.sp. 2 | 12b |
| **Typhloplanidae** |  |
| *Typhloplanidae* sp. 1 | 3 |
| *Typhloplanidae* sp. 2 | 12b |
| **Solenopharyngidae** |  |
| Solenopharyngidae. 1 | 1;12b |
| Solenopharyngidae sp. 2 | 12b |
| Solenopharyngidae sp. 3 | 12b |
| **Trigonostomidae** |  |
| *Ceratopera gracilis* (Graff, 1882) | 11a |
| *Trigonostomum australis* Willems, Artois, Vermin & Schockaert, 2004 | 11a |
| *Trigonostomum penicillatum* (Schmidt, 1857) | 5b |
| *Trigonostomum setigerum* Schmidt, 1852 | 3;5b;11a |
| *Trigonostomum venenosum* (Uljanin, 1870) | 3;5b |
| **Rhabditophora inc. sedis** |  |
| **Ciliopharyngiellidae** |  |
| *Ciliopharyngiella*?? sp. | 12b |

Refer to Table S1 for the identification of sampling stations.
