# Supplementary material for: Patterns of Diversity in Soft-Bodied Meiofauna: Dispersal Ability and Body Size Matter
Source: PLoS One. 2012 Mar 23;7(3):e33801. doi: 10.1371/journal.pone.0033801 (PMC3311549; doi:10.1371/journal.pone.0033801)
Supplement: Tables S6 — Gastrotricha. Species list and occurrence in Northern Sardinia. (DOC) [file pone.0033801.s007.doc]

**Table S6. Gastrotricha. Species list and occurrence in Northern Sardinia.**

| **Taxon** | **Station** |
| --- | --- |
| **Macrodasyida** |  |
| **Cephalodasyidae** |  |
| *Cephalodasys* n.sp1It. | 5a |
| *Cephalodasys* sp.2It | 12b |
| *Megadasys* n.sp. It | 5a |
| *Mesodasys laticaudatus* Remane, 1951 | 1;11b;12b |
| *Paradasys* n.sp. It | 12b |
| **Dactylopolidae** |  |
| *Dactylopodola typhle* (Remane, 1927) | 1;12b |
| **Lepidodasyidae** |  |
| *Lepidodasys unicarenatus* Balsamo, Fregni & Tongiorgi, 1994 | 1 |
| *Lepidodasys martini* Remane, 1926 | 1;12b |
| *Lepidodasys* n.sp. It | 12b |
| *Lepidodasys platyurus* Remane, 1927 | 12b |
| **Macrodasyidae** |  |
| *Macrodasys* n.sp. 1It | 1 |
| *Macrodasys n.*sp. 2It | 5a |
| *Urodasys apuliensis* Fregni, Faienza, Grimaldi, Tongiorgi & Balsamo, 1999 | 12b |
| *Urodasys viviparus* Wilke, 1954 | 1;4b;10;11b |
| *Urodasys* sp. 3It | 12b |
| **Planodasyidae** |  |
| *Crasiella* n.sp. It | 12b |
| **Thaumastodermatidae** |  |
| *Acanthodasys aculeatus* Remane, 1927 | 1;3;12b |
| *Acanthodasys* cf *caribbeanensis* Hochberg & Atherton, 2010 | 1 |
| *Diplodasys sanctaemariae* Hummon & Todaro, 2009 | 1;12b |
| *Oregodasys ocellatus* (Clausen, 1965) | 12b |
| *Ptychostomella mediterranea* Remane, 1927 | 1 |
| *Ptychostomella* n.sp. It | 12b |
| *Tetranchyroderma aapton* Dal Zotto, Ghiviriga & Todaro, 2010 | 12b |
| *Tetranchyroderma cirrophorum* Lévi, 1950 | 1;12b |
| *Tetranchyroderma heterotubulatum* Hummon, Todaro & Tongiorgi, 1993 | 1 |
| *Tetranchyroderma hirtum* Luporini, Magagnini & Tongiorgi, 1973 | 1;3;10 |
| *Tetranchyroderma inaequitubulatum* Todaro, Balsamo & Tongiorgi, 2002 | 1 |
| *Tetranchyroderma insulare* Balsamo, Fregni & Tongiorgi, 1994 | 4b |
| *Tetranchyroderma quadritentaculatum* Todaro, Balsamo & Tongiorgi, 1992 | 1 |
| *Tetranchyroderma symphorochetum* Hummon Todaro, Tongiorgi & Balsamo, 1998 | 1 |
| *Tetranchyroderma thysanogaster* Boaden, 1965 | 4b;10;11 |
| *Tetranchyoderma thysanophorum* Hummon, Todaro & Tongiorgi, 1993 | 5a;4b;11b |
| *Tetranchyroderma* n.sp. 1It | 1 |
| *Tetranchyroderma* sp. 2It | 1 |
| *Thaumastoderma mediterraneum* Remane, 1927 | 1 |
| **Turbanellidae** |  |
| *Paraturbanella dohrni* Remane, 1927 | 10 |
| *Paraturbanella pallida* Luporini, Magagnini & Tongiorgi, 1973 | 1;3;4b;11b;12b |
| *Paraturbanella teissieri* Swedmark, 1954 | 12b |
| *Paratubanella* n.sp. | 12b |
| **Incertae Sedis** |  |
| n.gen.1 n.sp**.** 1It | 12b |
| n.gen.2 n.sp. 1It | 12b |
| **Chaetonotida** |  |
| **Chaetonotidae** |  |
| *Aspidiophorus marinus* Remane, 1926 | 10 |
| *Aspidiophorus mediterraneus* Remane, 1927 | 10;11b |
| *Aspidiophorus paramediterraneus* Hummon, 1974 | 1;3;4b;11b;12b |
| *Aspidiophorus tentaculatus* Wilke, 1954 | 3 |
| *Chaetonotus aegilonensis* Balsamo, Todaro & Tongiorgi, 1992 | 12b |
| *Chaetonotus apechochaetus* Hummon, Balsamo & Todaro, 1992 | 1 |
| *Chaetonotus atrox* Wilke, 1954 | 1 |
| *Chaetonotus neptuni* Wilke, 1954 | 1;3;4b;12b |
| *Chaetonotus siciliensis* Hummon, Balsamo & Todaro, 1992 | 3;4b;12b |
| *Chaetonotus* n.sp. 1It | 1 1;3;10 |
| *Chaetonotus* n.sp. 2It | 2 12b |
| *Chaetonotus* n.sp. 3It | 3 12b |
| *Halichaetonotus euromarinus* Hummon & Todaro, 2010 | 1 |
| *Halichaetonotus* n.sp. It | 1 |
| *Heterolepidoderma* n.sp. It | 12b |
| **Muselliferidae** |  |
| *Musellifer delamarei* (Renaud-Mornant, 1968) | 1;12b |
| **Xenotriculidae** |  |
| *Heteroxenotrichula pygmaea* (Remane, 1934) | 9a |
| *Heteroxenotrichula* n.sp. It | 12b |
| *Xenotrichula punctata* Wilke, 1954 | 4b;11b |

Refer to Table S1 for the identification of sampling stations.
