# Supplementary material for: Patterns of Diversity in Soft-Bodied Meiofauna: Dispersal Ability and Body Size Matter
Source: PLoS One. 2012 Mar 23;7(3):e33801. doi: 10.1371/journal.pone.0033801 (PMC3311549; doi:10.1371/journal.pone.0033801)
Supplement: Tables S7 — Annelida. Species list and occurrence in Northern Sardinia. (DOC) [file pone.0033801.s008.doc]

**Table S7.** Annelida. Species list and occurrence in Northern Sardinia.

| **Taxon** | **Station** |
| --- | --- |
| **Nerillididae** |  |
| *Meganerilla* sp. | 13b |
| *Mesonerilla intermedia* Wilke, 1953 | 1;3;10;12b;13a |
| *Mesonerilla biantennata* Jouin, 1963 | 1;12b |
| *Mesonerilla armoricana* Swedmark, 1959* | 1;12b |
| *Mesonerilla* cf. *armoricana* Swedmark, 1959 | 13a, b |
| *Mesonerilla* n.sp. | 13b |
| *Nerillidium mediterraneum* Remane, 1928 | 1;3;4b;10;12b;13a |
| *Trochonerilla* sp. | 13b |
| **Polygordiidae** |  |
| *Polygordius* sp. | 13b |
| **Protodrilidae** |  |
| *Protodrilus* *gracilis* Von Nordheim, 1989 | 1,3,12b |
| *Protodrilus purpureus* (Schneider, 1868) | 13b |
| *Protodrilus similis* Jouin, 1970* | 4b,5a |
| **Psammodrilidae** |  |
| *Psammodrilus* n.sp | 1 |

*new to the Italian fauna; Refer to Table S1 for the identification of sampling stations.
