# Supplementary material for: Patterns of Diversity in Soft-Bodied Meiofauna: Dispersal Ability and Body Size Matter
Source: PLoS One. 2012 Mar 23;7(3):e33801. doi: 10.1371/journal.pone.0033801 (PMC3311549; doi:10.1371/journal.pone.0033801)
Supplement: Tables S8 — Rotifera. Species list and occurrence in Northern Sardinia. (DOC) [file pone.0033801.s009.doc]

**Table S8.**  Rotifera. Species list and occurrence in Northern Sardinia.

| **Taxon** | **Station** |
| --- | --- |
| **Monogononta** |  |
| **Brachionidae** |  |
| *Brachionus ibericus* Ciros-Peréz, Gómez & Serra, 2001 | 12c |
| *Brachionus urceolaris* Müller, 1773 | 12c |
| **Dicranophoridae** |  |
| *Encentrum* sp. | 6a;7; 12c |
| **Lecanidae** |  |
| *Lecane bulla* (Gosse, 1851) | 12c |
| **Lepadellidae** |  |
| *Colurella colurus* (Ehrenberg, 1830) | 6b;7 |
| *Colurella dicentra* (Gosse, 1887) | 6a |
| *Colurella* sp. | 8; 12c |
| *Lepadella* sp. | 7 |
| **Notommatidae** |  |
| *Eosphora ehrenbergi* Weber & Montet, 1918 | 12c |
| **Proalidae** |  |
| *Proales halophila* Remane, 1929 | 7;8;9b |
| *Proales similis* de Beauchamp, 1907 | 7 |
| *Proales* sp. | 6a;7;8; 12c |
| **Testudinellidae** |  |
| *Testudinella clypeata* (Müller, 1786) | 9b |
| *Testudinella obscura* Althaus, 1957 | 1 |
| **Bdelloidea** |  |
| **Philodinidae** |  |
| *Rotaria laticeps* Wulfert, 1942 | 12c |
| *Rotaria* sp. | 12c |

Refer to Table S1 for the identification of sampling stations..
